# Spontaneously evolved progenitor niches escape Yap oncogene addiction in advanced pancreatic ductal adenocarcinomas

**DOI:** 10.1038/s41467-023-37147-y

**Published:** 2023-03-15

**Authors:** Shigekazu Murakami, Shannon M. White, Alec T. McIntosh, Chan D. K. Nguyen, Chunling Yi

**Affiliations:** Lombardi Comprehensive Cancer Center, Georgetown University Medical Center, Washington, DC USA

**Keywords:** Tumour heterogeneity, Cancer stem cells, Pancreatic cancer

## Abstract

Lineage plasticity has been proposed as a major source of intratumoral heterogeneity and therapeutic resistance. Here, by employing an inducible genetic engineered mouse model, we illustrate that lineage plasticity enables advanced Pancreatic Ductal Adenocarcinoma (PDAC) tumors to develop spontaneous relapse following elimination of the central oncogenic driver - Yap. Transcriptomic and immunohistochemistry analysis of a large panel of PDAC tumors reveals that within high-grade tumors, small niches of PDAC cells gradually evolve to re-activate pluripotent transcription factors (PTFs), which lessen their dependency on Yap. Comprehensive Cut&Tag analysis demonstrate that although acquisition of PTF expression is coupled with the process of epithelial-to-mesenchymal transition (EMT), PTFs form a core transcriptional regulatory circuitry (CRC) with Jun to overcome Yap dependency, which is distinct from the classic TGFb-induced EMT-TF network. A chemical-genetic screen and follow-up functional studies establish Brd4 as an epigenetic gatekeeper for the PTF-Jun CRC, and strong synergy between BET and Yap inhibitors in blocking PDAC growth.

## Abstract (web summary)

It remains unclear how spontaneous Pancreatic Ductal Adenocarcinoma (PDAC) tumors escape Yap dependency. Here, the authors show that lineage plasticity promotes spontaneous relapse following YAP ablation and reveal key transcriptional drivers that overcome YAP addiction in PDAC.

## Introduction

The term “oncogene addiction” was first proposed 20 years ago to describe the phenomenon that cancer cells often exhibit exquisite dependencies on one or several oncogenic drivers to sustain tumor growth and progression^1^. While striking clinical responses have been achieved with drugs targeting driver oncogenes, nearly all tumor remissions are followed by eventual tumor relapse^2^. Thus, enumerating the mechanisms by which cancer cells adapt to oncogene suppression is essential for achieving sustained tumor control in patients^3^.

Kras is one of the most commonly mutated oncogenes whose activating mutations are detected in more than 20% of human cancers, including 95% of all pancreatic ductal adenocarcinoma (PDAC) cases^4^. Extensive studies using various genetically engineered mouse (GEM) models have firmly established the critical roles of oncogenic Kras in the initiation, progression, and maintenance of PDAC tumors^5–8^. Several Kras effector pathways have been implicated in PDAC initiation and maintenance including the mitogen-activated protein kinase (MAPK), phosphatidylinositol 3-kinase (PI3K), and Ral guanine nucleotide exchange factor (RalGEF) signaling pathways^9–12^. Further downstream, sustained oncogenic Kras signaling is known to induce extensive epigenetic and transcriptional reprogramming in neoplastic pancreatic epithelial cells, resulting in the progressive silencing of pancreatic lineage TFs, the gradual increase in lineage plasticity, and the activation of pleiotropic oncogenic TFs^13^.

Yap, which lacks a DNA-binding domain, partners with the Tead family of TFs to promote transcriptional programs that are central to the growth, survival, and therapy resistance in many types of solid tumors^14,15^. We and others have shown that Kras mutant PDAC tumors are highly addicted to Yap, which acts as a central transcriptional gatekeeper in maintaining the expression of the master metabolic TF Myc and other key metabolic genes, in promoting macropinocytosis during nutrient stress, and in orchestrating an immune suppressive tumor microenvironment (TME)^16–23^.

First-in-class Yap/Tead inhibitors have recently entered Phase I clinical trials for the treatment of advanced solid tumors with hyperactive Yap^24,25^. Unlike Myc whose inactivation impairs the development of the exocrine pancreas and the postnatal expansion of β-cells^26–28^, Yap is dispensable for pancreatic development and normal pancreatic functions^17,22^, potentially making it a safer drug target. Importantly, not only does Yap play a critical role in maintaining metabolic homeostasis and an immune suppressive TME in Kras mutant PDAC tumors, but Yap has also been identified as a major driver of advanced PDAC tumor relapse following the genetic ablation of oncogenic Kras or treatment by MAPK inhibitors or cytotoxic agents^15,29–31^. These results underscore the therapeutic potential of Yap inhibitors in the treatment of PDAC either as a single agent or in combination with other approved or experimental therapeutics.

Using an inducible, dual-recombinase GEM model^32^, we recently demonstrated that genetic deletion of Yap caused early pancreatic lesions to undergo full regression despite the presence of oncogenic Kras, but only temporarily blocked the growth of orthotopic tumors derived from poorly differentiated Kras:p53mutant PDAC cells^16^.

In the current study, we further explore the mechanisms of resistance to Yap ablation in spontaneous PDAC tumors and primary PDAC cells. Our study reveals the emergence of rare “progenitor” niches during the natural course of PDAC progression and illustrates that the PTFs form a core CRC with Jun to maintain oncogenic transcription in Yap-independent PDAC cells. Importantly, we find that BET inhibitors disrupt the PTF-Jun CRC and re-sensitizes PDAC cells to Yap blockade, illustrating the therapeutic potential of combining Yap and BET inhibitors for the treatment of PDAC.

## Results

### Following Yap ablation, a subset of advanced Kras:p53 mutant PDAC tumors develop spontaneous relapsed lesions with embryonic progenitor features

**Fig. 1:**
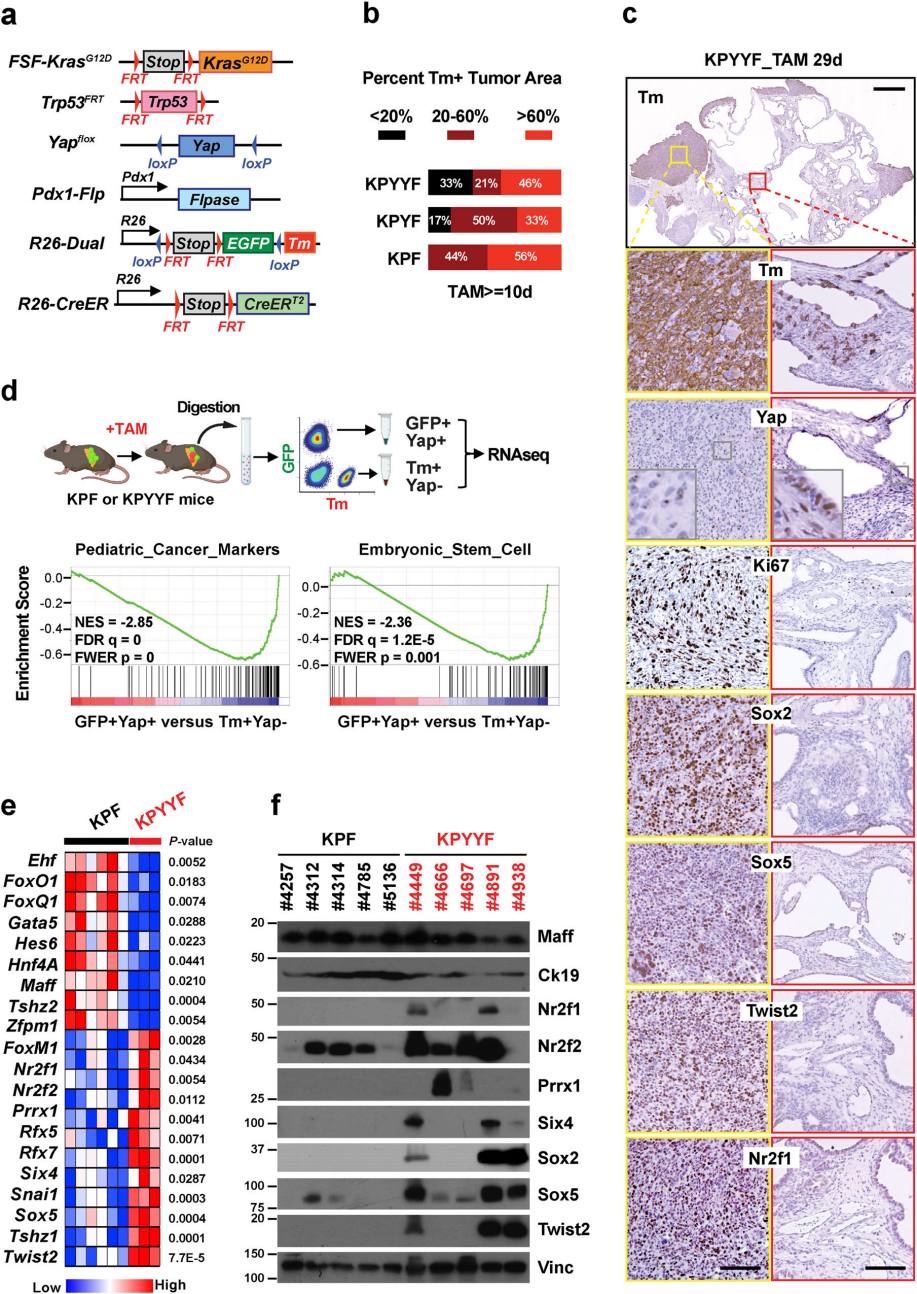
A subset of Kras:p53 mutant PDAC tumors develop spontaneous relapsed lesions with embryonic progenitor features following Yap ablation. **a** Genetic strategy to first activate *Kras*^*G12D*^ and delete *Trp53* in the pancreas via *Pdx1-Flp*, and subsequently delete *Yap* via TAM-induced Cre-loxP recombination systems. Note that *FSF-Kras*^*G12D*^*/Trp53*^*FRT*^ and *Yap*^*flox/flox*^ are under the separate controls of Flp and CreER, respectively. The *R26-Dual* reporter marks Flp-expressing KrasG12D cells with EGFP. Upon TAM-mediated CreER activation, the EGFP locus is removed, while tdTomato (Tm) is switched on. **b** The percentage of recombination evaluated by Tm+ area over Tm+ and GFP+ area based on IHC staining of KPF (*n* = 9), KPYF (*n* = 6) and KPYYF (*n* = 33) PDAC tumors following TAM treatment for at least 10 days. **c** Representative images of IHC analysis of indicated proteins in a KPYYF PDAC tumor after 29 days of TAM treatment. The top panel shows the overall Tm staining of an entire tumor with two small boxes marking a representative relapsing region (yellow) and a representative regressing region (red), respectively. The bottom panels include the higher-magnification IHC images of the indicated proteins at the marked regions. Scale bars indicate 1 mm (top) or 50 μm (all other panels). Experiments were performed on 13 different KPYY mice. **d** Experimental design (top) and the top two most enriched Gene Sets in Tm+/Yap− relative to GFP+/Yap+ cells according to GSEA (bottom) of RNA-seq of PDAC cells isolated from TAM-treated KPF and KPYYF mice. Unpaired, One-sided, *Z*-test. Created with BioRender.com. **e** Heatmap representing the expression of indicated genes in KPF (*n* = 6) and KPYYF (*n* = 3) tumors. Unpaired, two-tailed, Student’s *t*-test. **f** Western blot (WB) analysis of indicated proteins in KPF and KPYYF tumors. Vinc was used as the loading control. The mouse IDs belonging to the KPF or KPYYF cohorts are indicated. Shown is representative of three independent experiments. Source data are provided within a Source Data file.

We established large cohorts of *FSF-Kras*^*G12D/+*^*;Trp53*^*frt/+*^*;Yap*^*flox/flox*^*;Pdx-Flp;R26*^*FSF-CreER/Dual*^ (KPYYF) and *FSF-Kras*^*G12D/+*^*;Trp53*^*frt/+*^*;Pdx-Flp;R26*^*FSF-CreER/Dual*^ (KPF) mice as control (Fig. 1a). Since we previously found that YAP ablation halted the initiation and progression of early pancreatic lesions^16,17^, all mice were kept off tamoxifen (TAM) for the first two months after birth to allow PDAC tumors to develop in the presence of Yap. After the tumors became palpable, mice were put on a TAM-containing diet, which was expected to simultaneously turn off EGFP and switch on tdTomato (Tm) in the PDAC tumor cells of both KPF and KPYYF mice, and additionally delete Yap in KPYYF tumor cells (Fig. 1a). Consistent with the original report of this model^32^, 10 days of TAM treatment was sufficient to induce widespread Tm expression in KPF tumors according to immunohistochemistry (IHC) analysis (Fig. 1b, Supplementary Fig. 1a, b, Supplementary Table 1). In contrast, nearly 1/3 of the KPYYF tumors contained <20% Tm+ cells, even after prolonged TAM treatment far exceeding the 10-day window required for KPF tumors (Fig. 1b, Supplementary Fig. 1a, b, Supplementary Table 1). Furthermore, in nearly half of the KPYYF tumors that did exhibit widespread Tm activation, Yap remained expressed throughout the tumors, albeit at reduced levels (Fig. 1b, Supplementary Fig. 1a, b, Supplementary Table 1). Despite the frequent incomplete silencing of Yap, the KPYYF cohort exhibited a dramatically longer overall survival rate compared to the KPF cohort (Supplementary Fig. 1c). Moreover, a significantly higher percentage of Tm+ tumor areas stained positive for cleaved Caspase 3 (CC3) compared to the unrecombined GFP+Tm− tumor areas within KPYYF tumors or KPF tumors (Supplementary Fig. 1d). These observations strongly suggest that Yap suppression significantly reduces the fitness of most PDAC cells, causing them to be outcompeted and eliminated from some of the tumors.

Despite the apparent negative selection pressure against Yap-deficient PDAC cells, one-third of KPYYF mice developed localized, highly proliferative Tm+Yap− relapse nodules after extended TAM treatment (Fig. 1b, c, Supplementary Fig. 1a-b). Notably, these spontaneously developed Yap-independent nodules were generally poorly differentiated, and tended to be located around the peripheries of PDAC tumors (Fig. 1c, Supplementary Fig. 1b, e). RNA-seq of GFP+Yap+ and Tm+Yap− PDAC cells directly isolated from the relapsed nodules of TAM-treated KPF or KPYYF tumors revealed strong upregulation of markers of pediatric cancer, pluripotent/progenitor and embryonic stem cell, and epithelial to mesenchymal transition (EMT) along with Myc target genes in Tm+Yap− PDAC cells relative to GFP+Yap+ PDAC cells (Fig. 1d, Supplementary Fig. 2a, b). Conversely, genes involved in epithelial differentiation and cell–cell junction organization are significantly downregulated in Tm+Yap− PDAC cells compared to GFP+Yap+ PDAC cells (Supplementary Fig. 2b). Focused analysis of differentially expressed TFs showed that TFs associated with pluripotency and/or progenitor features (PTFs), including *Sox2*, *Sox5*, *Twist2*, *Six4*, *Nr2f1*, *Nr2f2*, and *Prrx1*^33–36^, are highly expressed in Tm+Yap− PDAC cells but barely detectable in GFP+Yap+ cells (Fig. 1e–g). In contrast, the levels of epithelial lineage TFs such as *Foxq1*, *Foxa2*, *Hnf4a, Ehf*, and *Gata4* were significantly downregulated in Tm+Yap− relative to GFP+Yap+ cells (Fig. 1e–g, Supplementary Fig. 1e).

Western blot (WB) analysis confirmed the upregulation of a subset of PTFs in YAP CRISPR knockout (KO) clones derived from human PDAC cell line Panc-1 (Supplementary Fig. 2c), indicating that expression of PTFs may also render human PDAC cells resistant to Yap loss. Finally, analysis of the publicly available TCGA and CPTAC human PDAC tumor RNAseq data^37,38^ revealed that human PDAC patients with tumors expressing high levels of PTFs and other genes upregulated in the relapsed nodules of TAM-treated KPYYF mice exhibited significantly worse overall survival compared to those expressing low levels of relapse-associated genes (Fig. 1d, Supplementary Fig. 2d). These results suggest that the same set of genes that allow murine PDAC tumors to adapt to Yap inactivation might also confer general therapeutic resistance in human PDAC patients.

### Advanced Yap-expressing murine and human PDAC tumors spontaneously develop “progenitor” niches that express PTFs

**Fig. 2:**
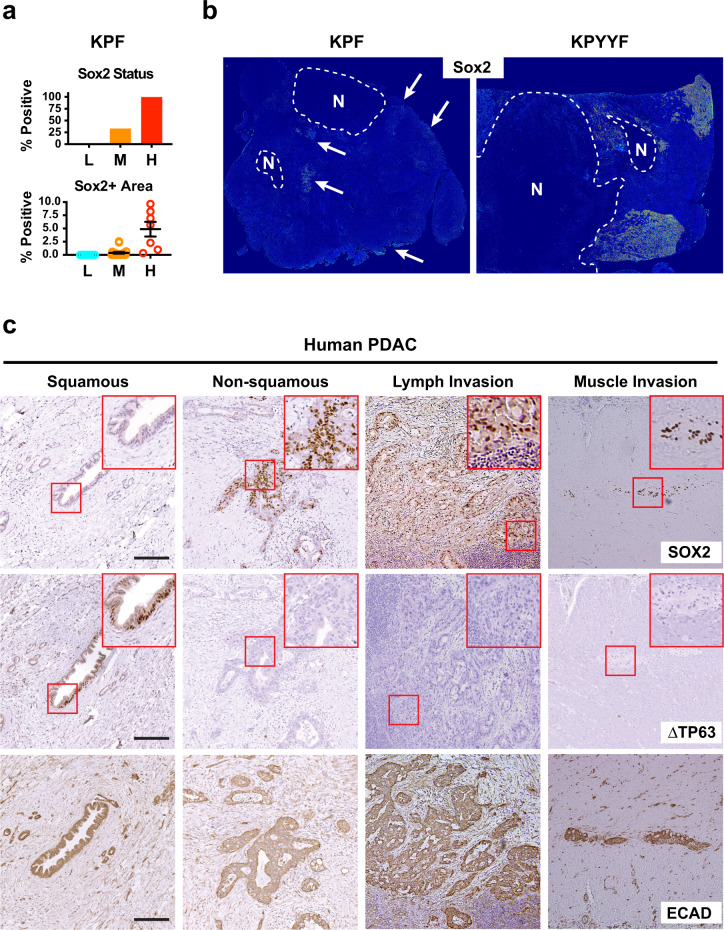
Advanced Yap-expressing murine and human PDAC tumors spontaneously develop “progenitor” niches that express PTFs. **a** Quantification of Sox2+ tumors (top) and Sox2+ tumor areas (bottom) in low (L, *n* = 7), intermediate (M, *n* = 21), and high (H, *n* = 7) grade primary PDAC tumors from KPF mice. Data are presented as mean value ± SEM. **b** Representative heatmaps from Sox2 IHC of TAM-treated KPF (*n* = 20) and KPYYF (*n* = 7) tumors. Arrows indicate isolated pockets of Sox2+ tumor cells in KPF tumors. Dashed lines demarcate necrotic regions (N). **c** Matched IHC images of indicated proteins at representative regions of primary human PDAC. Experiments were performed on five different hPDAC samples. Scale bars indicate 50 µm. Source data are provided within a Source Data file.

Our analysis revealed that upon TAM treatment about 1/3 of KPYYF tumors developed highly proliferative Tm+Yap− relapsed nodules hint to the existence of rare subpopulations of PDAC cells with a heightened ability to adapt to Yap loss. Indeed, IHC analysis of a large cohort of Yap+ KPF tumors revealed that while Sox2 and other PTFs were completely absent in low-grade tumor regions, a subset of intermediate-grade and 100% of high-grade KPC tumor regions contained clusters of malignant cells expressing Sox2 and other PTFs near some necrotic regions or invasive fronts (Fig. 2a, b, Supplementary Fig. 3a). Notably, Yap-independent relapsed lesions frequently arose from similar locations in KPYYF tumors after TAM treatment and tend to express high levels of nuclear Myc and Myc target genes (Figs. 1c, 2b, Supplementary Figs. 1e, 2b), suggesting that these pre-existing PTF-expressing niches, inherently resistant to stress due to their heightened plasticity, may expand in response to Yap ablation by activating Myc.

To determine whether human PDAC tumors could also spontaneously acquire PTF expression, we stained five surgically resected stage-3 PDAC tumors. In 3 of the 5 tumors, we detected small pockets of PDAC cells expressing SOX2, SOX5, and/or NR2F1 at various locations of the PDAC tumors (Fig. 2c, Supplementary Fig. 3b). While PTF+ murine or human PDAC cells exhibited similar levels YAP expression compared to adjacent PTF− cells, they were mostly negative for the squamous cell marker, deltaNp63^39,40^ (Fig. 2c, Supplementary Fig. 3d), implying that spontaneous activation of PTFs, likely uncoupled from YAP expression and squamous trans-differentiation, occur in both human and mouse PDAC cells during tumor progression.

### Acquisition of PTF expression is associated with EMT and resistance to Yap ablation

**Fig. 3:**
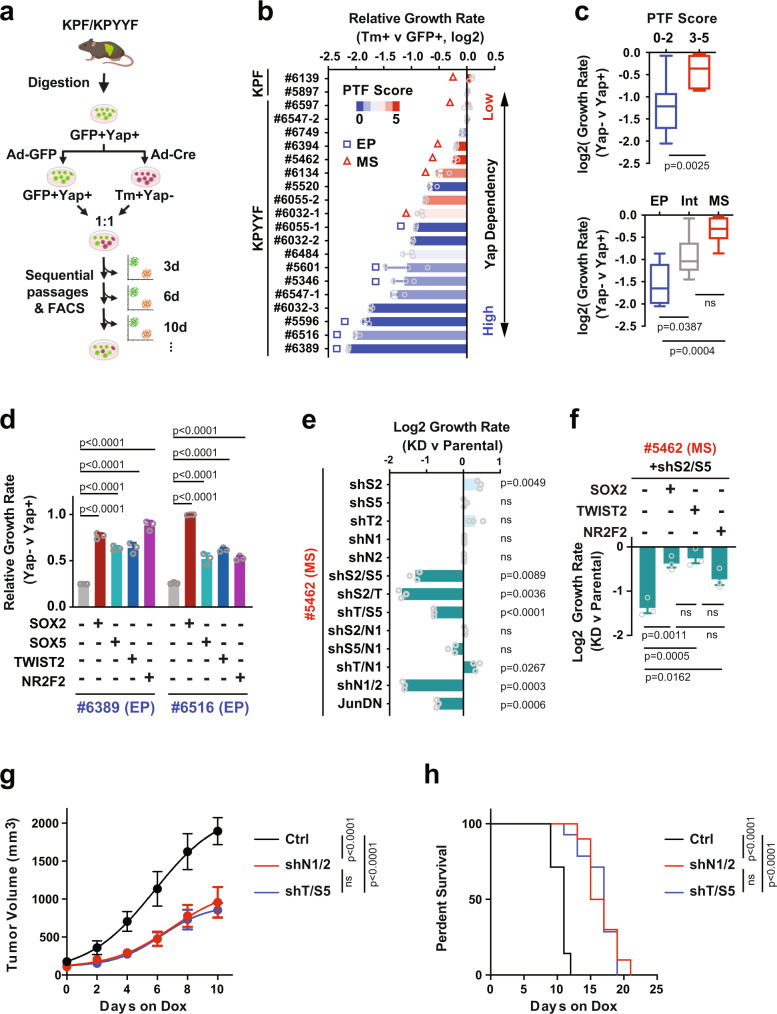
PTF expression is coupled with EMT and resistance to Yap ablation. **a** Illustration of the experimental design of ex vivo studies. Primary GFP+Yap+ PDAC lines isolated from KPF and KPYYF mice were infected with Ad-Cre or Ad-GFP. Equal numbers of GFP+Yap+ and Tm+Yap− PDAC cells were mixed for sequential passaging and FACS analysis every 3–4 days. Created with BioRender.com. **b** Log2 relative growth rate of Tm+ cells relative to GFP+ cells within the same co-cultures (*n* = 3 independent experiments). The PTF scores are indicated by color scales. PDAC cells classified as epithelial (EP) or mesenchymal (MS) differentiation based on Ecad expression and localization are marked by rectangles (EP) and triangles (MS), respectively. **c** Log2 relative growth rates of Tm+Yap− relative to GFP+Yap+ cells of KPYYF lines grouped by PTF scores or differentiation status. Top panel: *n* = 12 and 7 independent cell lines for the Low (0–2) and High (3–5) PTF groups, respectively. Centre at the median, box bounds 25th and 75th percentiles, whiskers minima and maxima. Unpaired, two-tailed, Student’s *t*-test. Bottom panel: *n* = 6, 7, and 6 independent cell lines from the EP, Intermediate (Int), and MS groups, respectively. One-way ANOVA with Tukey’s multiple comparisons. **d** Relative growth rates (Tm+Yap− versus GFP+Yap+) in two EP PDAC lines (#6516, #6389) expressing a single indicated exogenous PTF relative to the respective parental controls. *n* = 3. One-way ANOVA with Tukey’s multiple comparisons. **e** Log2 relative growth rate of the indicated single or double PTF-KD relative to parental PDAC cells derived from an MS tumor #5462 (*n* = 3 independent experiments). Unpaired, two-tailed, Student’s *t*-test. **f** Log2 relative growth rate of Sox2/Sox5 double KD #5462 cells with or without expressing indicated exogenous PTF relative to parental control (*n* = 3 independent experiments). One-way ANOVA with Tukey’s multiple comparisons. **g** Changes in tumor volumes of subcutaneous tumors derived from #5462 cells carrying Dox-inducible shNr2f1/Nr2f2 (shN1/N2; *n* = 10 mice), shTwist2/Sox5 (shT/S5; *n* = 15 mice), or vector control (Ctrl; *n* = 7 mice) after indicated days of Dox treatment. Two-way ANOVA test with multiple comparisons. **h** Kaplan–Meier survival curve of mice carrying Ctrl (*n* = 7), shN1/N2 (*n* = 10), and shTwist2/Sox5 (*n* = 15). Mantel–Cox Long Rank test. Data are presented as mean value ± SEM. Source data are provided within a Source Data file.

To further interrogate the relationship between Yap dependency and PTF expression, we established cultures from 19 KPF and KPYYF primary PDAC tumors that were either untreated or failed to exhibit robust recombination after exposure to TAM (Fig. 3a). Immunofluorescence (IF), IHC and WB analysis showed that the primary PDAC cultures largely retained the differentiation status of the original PDAC tumors, and could be divided into three main groups based on the expression and subcellular localization patterns of Ecad and classic mesenchymal TFs Zeb1 and Slug: (1) the epithelial (EP) type defined by high membrane Ecad and lack of expression in mesenchymal TFs Zeb1 and Slug, (2) the intermediate (Int) type characterized by low and mostly cytoplasmic Ecad and sporadic Zeb1/Slug expression, and (3) the mesenchymal (MS) type expressing Zeb1 and Slug but not Ecad (Supplementary Figs. 4, 5a). In parallel, we measured the expression of Yap, Sox2, Sox5, Twist2, Nr2f1, and Nr2f2 by WB, and assigned each primary PDAC line with a PTF score according to the numbers of highly expressed PTFs (Supplementary Fig. 5a). Consistent with our in vivo observations, while PTF scores were not linked to Yap expression levels, they were strongly correlated with the differentiation statuses, with all 5 MS lines exhibiting PTF scores of 4 or 5 and all 8 EP lines exhibiting PTF scores of 0 or 1 (Supplementary Figs. 4, 5a).

To assess how differentiation status and PTF scores influence the dependency of PDAC cells on Yap, we devised a quantitative Yap-dependency assay by infecting each primary PDAC line separately with Ad-GFP and Ad-Cre, followed by mixing equal ratios of Ad-GFP-treated GFP+ and Ad-Cre-treated Tm+ PDAC cells and performing sequential flow cytometry (FC) analysis to compare their relative proliferation rates within the same cultures (Fig. 3a, Supplementary Fig. 5a, b). As expected, there was no difference in proliferation between GFP+ and Tm+ populations derived from KPF cells, given that Yap expression was not affected by Cre treatment (Fig. 3b, Supplementary Fig. 5a). In contrast, the changes in the ratios of Tm+Yap− and GFP+Yap+ cells over time varied dramatically among the different KPYYF lines (Fig. 3b, Supplementary Fig. 5b). Overall, we found that EP lines with low PTF scores exhibited significantly higher dependency on Yap compared to intermediate and MS lines with high PTF scores, which was validated by 2D and 3D colony formation assays of representative EP and MS lines (Fig. 3b, c, Supplementary Fig. 5a–c). Similarly, according to the DepMap CRISPR screen^41^, human PDAC lines with high PTF scores exhibited significantly lower dependency on YAP compared to those with low PTF scores (Supplementary Fig. 5d). Interestingly, PTF-high and low human PDAC cells did not show differential dependency on YAP paralogue TAZ (Supplementary Fig. 5d). Among the primary murine PDAC cells, Taz expression levels were highly variable, and did not correlate with Yap dependency, differentiation status or PTF scores (Supplementary Fig. 5a).

Together, these results support the notion that spontaneous acquisition of PTF expression during the natural course of PDAC tumor evolution, unrelated to Yap or Taz expression, could potentially enable a subset of PDAC tumor cells to overcome their addiction to Yap.

### Expression of individual PTFs is sufficient to overcome Yap dependency

To directly test how the expression of individual PTFs affects the dependency of PDAC cells on Yap, we stably expressed SOX2, SOX5, TWIST2, or NR2F2 in #6389 and #6516—two of the primary PDAC lines with low PTF scores and high Yap dependency (Fig. 3b, Supplementary Fig. 5a–c, e). These PTF-reconstituted lines alongside their parental controls were treated with Ad-Cre or Ad-GFP, and subjected to Yap-dependency assay as outlined in Fig. 3a. While the ratios of Tm+Yap− relative to GFP+Yap+ cells derived from parental cells rapidly decreased, they remained largely steady or slightly decreased over time in PTF-reconstituted cells (Fig. 3d). These results indicate that exogenous expression of a single PTF is sufficient to overwrite, at least partially, the dependency of PDAC cells on Yap.

### PTF expression is required for maintaining the growth of Yap-independent PDAC cells in vitro and in vivo

Next, we examined the roles of PTFs in maintaining the proliferation of PDAC cells with high PTF scores and low Yap dependency. To this end, we introduced Dox-inducible, fluorescently labeled shRNAs targeting a single or a pair of the PTFs into #5462 cells, a primary PDAC line that exhibited high expression of all five PTFs associated with Yap independency (Fig. 3b, c, Supplementary Fig. 5a). Sequential FC analysis of co-cultures of parental and knockdown (KD) #5462 cells in the presence of Dox showed that while KD of a single PTF had no effect on cell proliferation, certain combinational KD of two different PTFs significantly reduced the proliferation of #5462 cells (Fig. 3e), which could be rescued by overexpressing a single PTF (Fig. 3f). Finally, we confirmed that KD of two different PTFs also significantly inhibited tumor growth rate and prolonged survival in vivo (Fig. 3g, h). Together, these results indicate that PTFs function in a partially overlapping fashion to maintain the growth of Yap-independent PDAC tumor cells in vitro and in vivo.

### Acquisition of PTF expression and Yap independency is associated with global epigenetic reprogramming

Based on our observations that PTF-expressing cells tended to first emerge near necrotic regions or invasive edges of the advanced murine and human PDAC tumors (Fig. 2, Supplementary Fig. 3a, b), we hypothesized that epigenetic reprogramming induced by p53 inactivation and stresses within the TME may cause the spontaneous re-activation of PTFs in subpopulations of PDAC cells. To test this hypothesis, we subjected PTF-low #6389 and #6516 cells to 2 weeks of treatment with inhibitors targeting epigenetic enzymes involved in gene silencing (DNMT1 inhibitor 5’-Aza or EZH2 inhibitor DZNep), nutrient deprivations, or hypoxia-mimetic agent CoCl_2_, and performed IF analysis on Sox2, Sox5, and Twist2. While the effects were highly variable, the majority of stress conditions tested induced various degrees of activation of at least one of the three PTFs in either or both of the cell lines (Supplementary Fig. 6). Importantly, 2 weeks of exposure to 5’-Aza and/or DZNep also induced robust activation of Sox2, Sox5 and/or Twist2 treatments under full nutrient conditions (Supplementary Fig. 6), confirming the involvement of epigenetic reprogramming in activating PTFs.

**Fig. 4:**
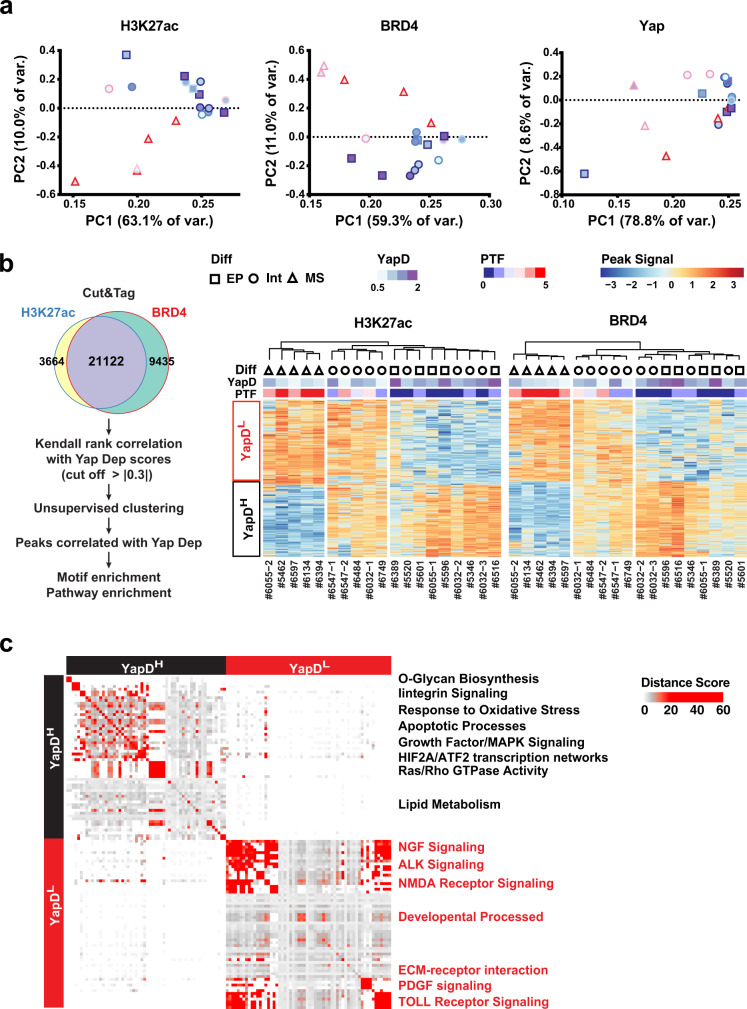
Acquisition of PTF expression and Yap independency is associated with global epigenetic reprogramming. **a** PCA plot segregating all 19 primary murine PDAC lines according to the global H3K27ac, BRD4, and Yap Cut&Tag signal profiles. The differentiation statuses (Diff) of PDAC lines are represented by shapes (EP: square; Int: circle; MS: triangle). The relative Yap dependency (YapD) scores are indicated by colors filling the shapes. The PTF scores are marked by the line colors of the shapes. **b** Analysis workflow (left) and heatmap depicting unsupervised clustering (right) of all 19 primary PDAC lines according to the signals from overlapping H3K27ac and BRD4 Cut&Tag peaks whose cross-sample signal rank variations significantly (|corr| > 0.3) correlate with high (YapD^H^) or low (YapD^L^) Yap dependency based on Kendall rank correlation. The differentiation statuses, Yap dependency, and PTF scores are depicted by the same schemes as (**a**). **c** Heatm**a**p representing the distance scores of significantly enriched pathways or biological processes mapped to YapD^H^ or YapD^L^ peaks from **b** as determined by GREAT GeneSetCluster analysis. Source data are provided within a Source Data file.

To further assess how PTF expression may affect the global epigenetic landscape and Yap chromatin binding, we performed H3K27ac, Brd4, and Yap Cut&Tag with all 19 primary PDAC lines. Unsupervised clustering and principal component analysis (PCA) revealed that the overall signal profiles of H3K27ac and Brd4, which mark active enhancers and promoters, clearly segregated mesenchymal PDAC cells with high PTF and low Yap dependency scores from epithelial or intermediate PDAC cells with low PTF and high Yap dependency scores (Fig. 4a, Supplementary Fig. 7a). In contrast, overall Yap binding performed much worse in distinguishing the different types of PDAC cells (Fig. 4a, Supplementary Fig. 7a).

To identify genomic loci associated with changes in Yap dependency, we performed Kendall correlations of Yap dependency scores across all 19 PDAC lines against the corresponding H3K27ac and Brd4 signals at each of the cis-regulatory elements (CREs) and transcription start sites (TSSs) that are active in at least 3 PDAC lines as indicated by their positivity for both H3K27ac and Brd4 (Fig. 4b). As expected, Rank-Rank Hypergeometric Overlap (RRHO) analysis^42^ showed strong concordance between H3K27ac and Brd4 in their correlation co-efficiencies against Yap dependency scores (Supplementary Fig. 7b). Using a cutoff of ±0.3, we identified 264 and 276 sites whose H3K27ac and Brd4 signals correspond to high and low Yap dependencies, respectively (Fig. 4b). Unsupervised clustering of either H3K27ac or Brd4 signals at these 540 sites segregated the 19 PDAC lines into three major groups, with cluster 1 containing mesenchymal, PTF-high and Yap-independent PDAC cells, cluster 2 containing PDAC cells with intermediate differentiation, PTF and Yap dependency statuses, and cluster 3 containing epithelial PDAC cells with low PTF expression and high Yap dependency (Fig. 4b). To test whether acquisition of Yap independency is associated with loss of Yap binding, we assessed Yap binding at the 540 CREs and TSSs whose activities associated with high or low Yap dependencies. Unexpectedly, we found that 73% of the CREs and TSSs associated with reduced Yap dependency were bound by Yap in at least three PDAC lines, compared to 59% of the regions associated with high Yap dependency (Supplementary Fig. 7c). Unsupervised clustering of Yap signals at these sites further revealed that variations in Yap binding strengths across PDAC cell lines largely tracked those of Brd4 and H3K27ac (Fig. 4b, Supplementary Fig. 7d), suggesting global epigenetic reprogramming associated with PTF expression and EMT rather than a loss in Yap binding is likely responsible for reducing the dependency of PDAC cells on Yap.

Squamous (also known as basal) trans-differentiation has been linked to worse outcomes in human PDAC patients^43–45^. Using Brd4 signals at TSSs as surrogates for gene expression, we performed unsupervised clustering of the 19 murine PDAC lines against a PDAC squamous signature defined by Somerville et al.^46^, which segregated the 19 primary murine PDAC lines into a squamous-high and a squamous-low groups (Supplementary Fig. 7e). Consistent with the observations from IHC analysis of human PDAC tumors (Fig. 2c, Supplementary Fig. 3d), the squamous-high group was not enriched with PDAC cells with high PTF and low Yap dependency scores (Supplementary Fig. 7e). The PDAC squamous signature also failed to separate human PDAC cells with high Yap dependency from those with low Yap dependency (Supplementary Fig. 7f), further confirming that acquisition of PTF expression and Yap independency is likely to independent of squamous differentiation.

Using Genomic Regions Enrichment of Annotations Tool (GREAT)^47^, we identified the genes located in the vicinity of the 540 enhancers and promoters linked to Yap dependency. Gene Ontology (GO) enrichment analysis indicated that genes involved in O-glycan biosynthesis, integrin signaling, response to oxidative stress, apoptotic processes, growth factor/MAPK signaling, HIF2A/ATF2 transcription networks, Ras/Rho GTPase activity, and lipid metabolism are significantly over-represented in genomic regions highly active in Yap dependent PDAC cells, whereas genomic regions specifically activated in Yap-independent PDAC cells are enriched for genes associated to NGF signaling, ALK signaling, NMDA receptor signaling, developmental processed, ECM–receptor interaction, PDGF signaling, and TOLL receptor signaling (Fig. 4c).

### PTFs and Jun co-occupy active enhancers and promoters associated with Yap independency

Differential Hypergeometric Optimization of Motif EnRichment (HOMER) analysis^48^ revealed that the homeo domain (HD) and nuclear receptor (NR) motifs recognized by the master epithelial lineage TFs HNF1b and HNF4a^49–51^, respectively, are preferentially enriched at the enhancers and promoters associated with Yap dependency (Supplementary Fig. 8a). In contrast, motifs recognized by the AP-1 (bZIP), Twist (bHLH), Smad (MAD) and Sox (HMG) family of TFs are significantly more prevalent at the enhancers and promoters associated with Yap independency (Supplementary Fig. 8a). Expression of a dominant-negative Jun mutant (JunDN), which blocks all members of the AP-1 family of TFs, significantly reduced the proliferation of primary PDAC cells in Yap-independent #5462 cells as well as Yap-dependent #6389 and #6516 cells (Fig. 3e, Supplementary Fig. 8b).

**Fig. 5:**
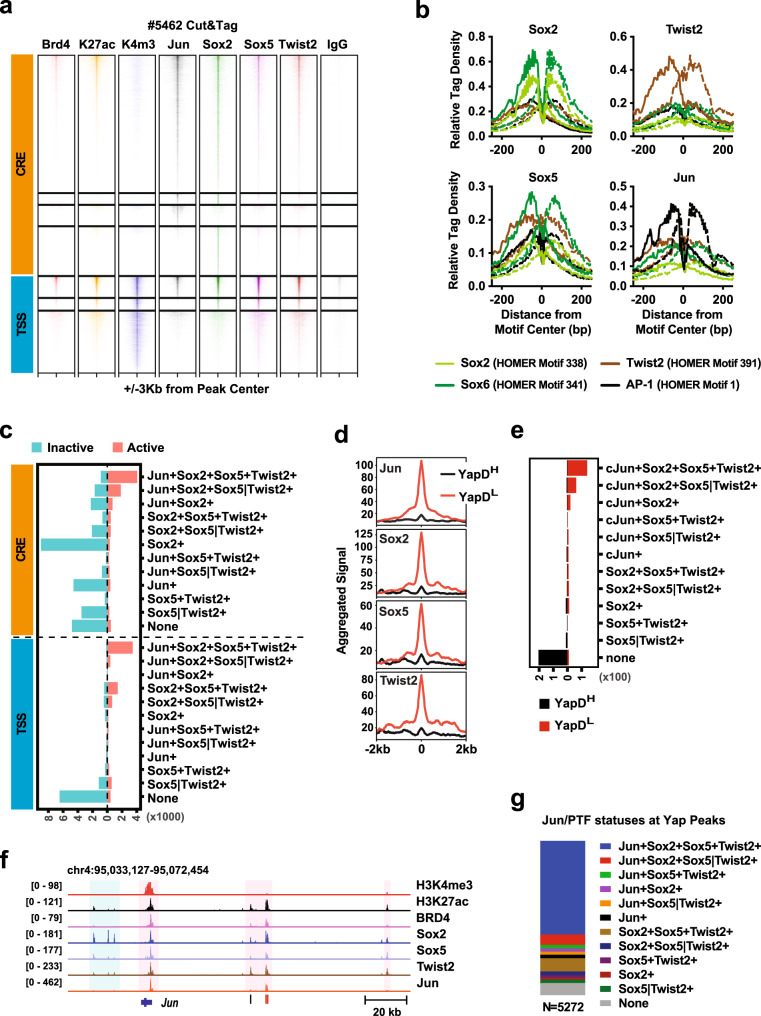
PTFs and Jun co-occupy active enhancers and promoters associated with Yap independency. **a** Cut&Tag signal heatmaps from the indicated antibodies within ±3kb from the centers of merged CRE and TSS sites bound by at least one of the TFs or histones in #5462 cells. TSS sites are defined by H3K4me3 positivity. **b** Relative Tag Density from Cut&Tag analysis of #5462 cells with indicated antibodies at ±250 bp from the centers of the indicated HOMER motif sites. **c** Summary of the numbers of active (light red) and inactive (light green) CRE and TSS sites with different binding statuses by Jun, Sox2, Sox5, and/or Twist2 in #5462 cells. Active CRE and TSS are defined by H3K27ac and BRD4 double positivity. Sox5|Twist2+ indicates peaks bound by either Sox5 or Twist2, but not both. **d** Aggregated Jun, Twist2, Sox2, and Sox5 Cut&Tag signals within ±2 kb from the centers of BRD4 peaks associated with YapD^H^ or YapD^L^ in #5462 cells. **e** Summary of the numbers of genomic loci associated with YapD^H^ (black) and YapD^L^ (red) with different binding statuses by Jun, Sox2, Sox5, and Twist2 in #5462 cells. **f** Genomic tracks showing the distributions of Cut&Tag peaks from indicated antibodies surrounding the *Jun* gene in #5462 cells. **g** Summary of the percentages of Yap peaks with different binding statuses by Jun, Sox2, Sox5, and Twist2 in #5462 cells. Source data are provided within a Source Data file. Cut&Tag data are deposited in the GEO database under accession code GSE224566.

To determine how PTFs may cooperate with AP-1 TFs in directing epigenetic reprogramming in PDAC cells, we performed Sox2, Sox5, Twist2, and Jun Cut&Tag in Yap-independent #5462 cells that express high levels of PTFs and Jun, and additionally in #6547-1 cell with intermediate Yap dependency and expressing lower levels of PTFs and Jun. Confirming the specificity of the antibodies used, all four TFs exhibited marked higher overall chromatin-binding in #5462 cells relative to #6547-1 cells in accordance with their relative expression levels (Supplementary Fig. 8c), and specific enrichment of read densities around their respective canonical motif sites (Fig. 5b, Supplementary Table 2). Next, we investigated the extent of overlaps among the TFs, and the statuses of H3K4me3, H3K27ac, and BRD4 at each site bound by one or more of the four TFs in #5462 cells. Based on the well-established role of H3K4me3 in demarcating TSSs, we divided the combined 57,873 unique genomic sites recognized by at least one of the antibodies into H3K4me3^+^ TSS and H3K4me3^−^ cis-regulatory elements, CRE (Fig. 5a, c). Out of the total 17,552 TSS peaks and 40,321 CRE peaks, 7714 (44%) TSS and 9587 (24%) CRE sites were positive for both H3K27ac and BRD4 and therefore most likely involved in active transcription (Fig. 5a). Remarkably, among the active TSS and CRE sites, 45–26% and 42–26% were co-occupied by all four or three of the TFs analyzed, respectively (Fig. 5a, c, Supplementary Fig. 8d). In contrast, only 2–6% inactive TSS and 3–8% inactive CRE sites were bound by at least three TFs, whereas >90% of unique Jun, Sox2, Sox5, or Twist2 peaks coincided with inactive CRE sites (Fig. 5a, c, Supplementary Fig. 8d). The active TSS and CRE sites bound by at least three of the TFs analyzed encompassed 76% of the genomic loci associated with low Yap dependency, whereas 79% of the genomic loci associated with high Yap dependency were bound by none of the four TFs in #5462 cells (Fig. 5d, e). CREs occupied by all four TFs were strongly enriched at SE regions, representing nearly 40% of all CREs located within SE regions (Supplementary Fig. 8e). Finally, among the 5272 Yap peaks detected in #5462 cells, 4858 (92%) were co-bound by at least one of the Jun/PTFs, with 3201 (61%) bound by all four TFs (Fig. 5g). These Cut&Tag data along with the functional studies from above strongly suggest that PTFs and Jun function cooperatively to maintain activate transcription and reduce the dependency of PDAC cells on Yap to maintain transcriptional homeostasis.

### PTFs form a CRC with Jun in Yap-independent PDAC cells

To determine how PTFs and Jun are recruited to and maintain active CREs in Yap-independent PDAC cells, we performed differential HOMER analysis comparing transcriptionally active and inactive CRE sites bound by each TF, which identified the AP-1 motif as the most over-represented motif at the active sites of all four TFs (Supplementary Fig. 8f, g). Similar results were obtained using TOBIAS (Transcription factor Occupancy prediction By Investigation of ATAC-seq Signal), a computational framework that detects the so-called TF “footprints”—short DNA sequences protected from Tn5 tagmentation by TFs bound at these sites (Supplementary Fig. 9a)^52^.

**Fig. 6:**
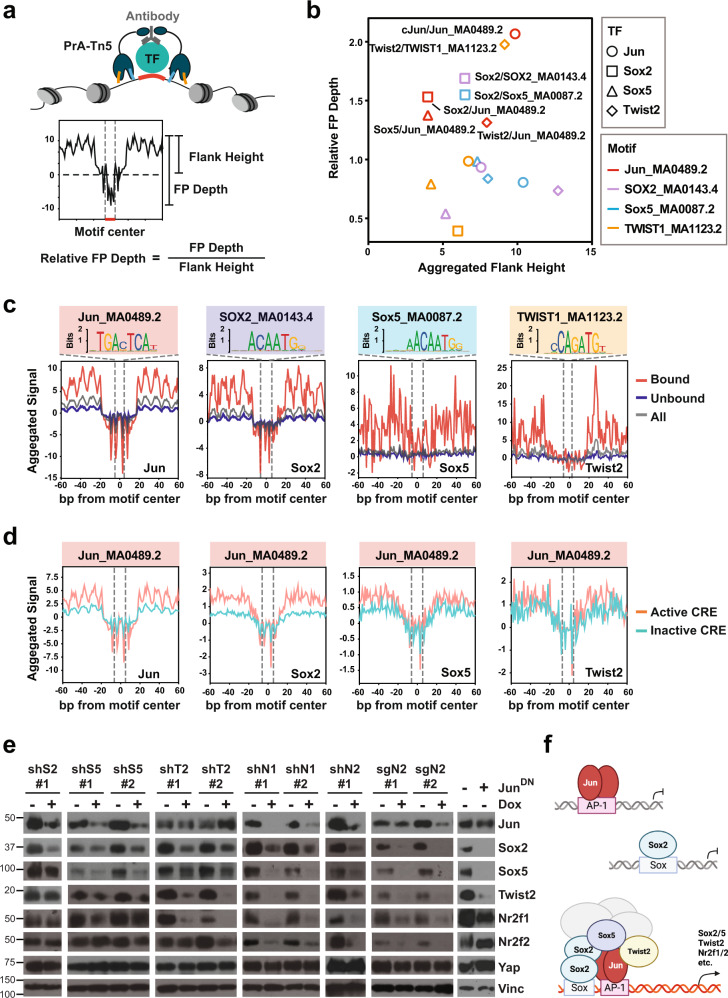
PTFs and Jun form a CRC with Jun acting as the dominant chromatin anchor in Yap-independent PDAC cells. **a** Schematic illustration showing chromatin-bound TF (green) recognized by its antibody (gray), which in turn recruits ProteinA-Tn5 (PrA-Tn5: dark blue) to tagment the accessible chromatin regions in the vicinity. The motif (red) bound by the TF is protected from tagmentation, leaving a footprint (FP) whose relative depth is calculated by dividing the absolute FP Depth by Flank Height (the height of peak signals immediately flanking the motif over the background). Created by S.M. **b** Scatter plot representing Relative FP Depths and the corresponding Aggregated Flank Heights of the indicated TFs at the sites matching the indicated motifs. TFs are represented by shapes (Jun: circle; Sox2: square; Sox5: triangle; Twist2: diamond). JASPAR motifs are presented by line colors (Jun_MA0489.2: red; SOX2_MA0143.4: purple; Sox5_MA0087.2: blue; TWIST1_MA1123.2: orange). **c** Aggregated signals from bound (red), unbound (blue), or all (gray) Jun, Sox2, Sox5, and Twist2 peaks centered on the indicated motif (marked by gray dashed lines). The total numbers of bound, unbound and all peaks for each antibody are Jun: 2785, 7731, and 10516; Sox2: 553, 2279, and 2832; Sox5: 127, 1452, and 1579, and Twist2: 230, 1078, and 1308. **d** Aggregated signal centering the Jun_MA0489.2 motif (marked by gray dashed lines) within the active (pink) or inactive (green) CRE sites bound by Jun, Sox2, Sox5, or Twist2. **e** Western blot analysis of indicated proteins in #5462 cells carrying Dox-inducible shRNAs or sgRNAs targeting indicated PTFs or constitutively overexpressing a dominant negative Jun mutant (Jun^DN^) in the presence or absence of Dox. Vinc was used as the loading control. Shown is representative of three independent experiments. **f** A working model of cooperative binding of Jun and PTFs to AP-1 sites that drive active transcription of target genes. Created with BioRender.com. Source data are provided within a Source Data file. Cut&Tag data are deposited in the GEO database under accession code GSE224566.

“Flanking accessibility” and “Footprint depth” are two major parameters commonly used to character TF footprints^52,53^. The “Flanking accessibility”, which measures the relative transposition frequencies immediately adjacent to the motifs, reflecting the amount of TF present around the sites (Fig. 6a). On the other hand, the “Footprint depth” assesses the relative protection of the motif sequences from transposition, indicative the binding strength of TF at the sites (Fig. 6a). Using the Tn5-bias-corrected footprint profiles generated by TOBIAS, we first calculated the “Flank height”, which is equivalent to “Flanking accessibility”, and “Relative footprint depth” (“Footprint depth” divided by “Flank height”) from aggerated footprints of each TF bound to its putative binding sites (Fig. 6b, c). As expected, Jun and Sox2 impinged strong, well-defined footprints at their canonical motifs, as indicated by both high flank accessibility and strong protection of the core motif sequences (Fig. 6b, c). In contrast, Sox5 did not leave clear footprints at the predicted Sox5-binding sites, whereas Twist2 imposed relatively shallow footprints implying weak binding at its putative binding sites (Fig. 6b, c). Given the extensive overlaps in chromatin binding among Jun and the PTFs (Fig. 5c, d), we investigated whether Jun and the PTFs could be recruited in trans to each other’s binding sites. Strikingly, even though all four TFs exhibited direct binding to their canonical motifs (Fig. 5b), only Jun recruited other PTFs to its direct binding sites as evidenced by the clear footprints matching AP-1 sites detected within Sox2, Sox5, and Twist2 peaks, especially among those corresponding to active CREs and/or overlapping Jun peaks (Fig. 6b–d, Supplementary Fig. 9b). Conversely, the co-recruitment of PTFs strongly enhanced the binding of Jun to AP-1 sites, as indicated by an increase in both flank accessibility and footprint depth of Jun footprints (Supplementary Fig. 9c). Similarly, the co-recruitment of PTFs and Jun also increased the binding strengths of Sox2 and to lesser extent Twist2 to their canonical sites (Supplementary Fig. 9c).

The frequent co-occupancy of Jun and PTFs at active CREs especially within SE regions and their functional importance in maintaining in vitro and in vivo growth indicate that they may form an autoregulatory loop with each component promoting the expression of itself and others—known as CRC^54^. WB analysis of #5462 cells with knockdown of individual PTFs or overexpression of a dominant-negative Jun mutant showed that inhibition of a single PTF or Jun is sufficient to reduce the expression of the others (Fig. 6e), thus confirming their ability to promote each other’s expression. Interestingly, although both PTFs and other classic EMT-TFs are expressed in Yap-independent PDAC cells, classic EMT-TFs but not PTFs were substantially induced in PDAC cells that underwent TGFb-induced morphological EMT (Supplementary Fig. 9d, e).

Taken together, our analysis supports a model that during PDAC progression, CREs that lessen the dependency of PDAC cells on Yap are first primed by AP-1 TFs binding directly to their canonical binding sites; subsequently, the PTFs are recruited to AP-1 sites and adjacent Sox and to lesser extent Twist sites, which stabilize the AP-1/PTF transcriptional complexes and potentiate the progressive activation of these loci and downstream target genes (Fig. 6f).

### Bromodomain and extra-terminal motif inhibitors (BET-i) block the expression of PTFs and sensitize PDAC cells to Yap inhibition

**Fig. 7:**
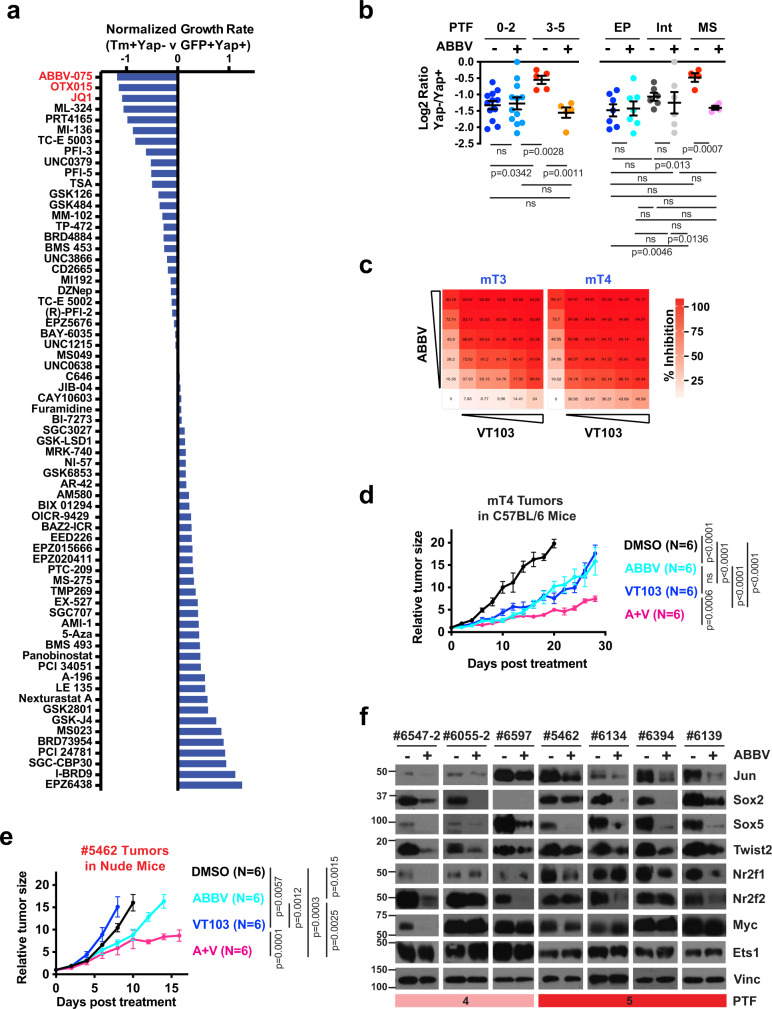
BET inhibitors block the expression of PTFs and sensitize PDAC cells to Yap inhibition. **a** Log2 relative growth rates of #5346 PDAC cells pre-treated with Ad-Cre (Tm+Yap−) or Ad-GFP (GFP+Yap+) co-cultured over a 2-month period in the presence of indicated epigenetic inhibitors normalized to DMSO. BET inhibitors are highlighted in red. **b** Log2 relative growth rates of Yap− relative to Yap+ cells in primary PDAC lines treated for 6 days with BET inhibitor ABBV-075 (+) or DMSO control (−). Primary PDAC lines are grouped based on PTF score (left) and differentiation status (right). Left panel: *n* = 12 and 5 independent cell lines for the Low (0–2) and High (3–5) PTF groups, respectively. Right panel: *n* = 7, 6, and 4 for EP, Int, and MS groups, respectively. Unpaired, two-tailed, Student’s *t*-test. Data are presented as mean value ± SEM. **c** Dose−response matrixes generated by SynergyFinder of ABBV plus Yap/Tead inhibitor VT103 in mT3 (Loewe synergy score = 31.7) and mT4 (Loewe synergy score = 30.7) PDAC cells derived from *KrasG12D/+:Trp52R172H/+:Pdx-Cre* (KPC) mice. **d**, **e** Changes in relative tumor size of mT4 (**d**) and #5462 (**e**) xenografts treated with ABBV (light blue), VT103 (blue), ABBV + VT103 (pink), or DMSO (black). *N* = 6 mice for each treatment arm. Data are presented as mean value ± SEM. Two-way ANOVA test. **f** Western blot analysis of indicated proteins in the indicated PTF-expressing primary PDAC lines treated with ABBV or DMSO. Vinc was used as the loading control. Shown is representative of three independent experiments. Source data are provided within a Source Data file.

In addition to the TFs, the process of epigenetic reprogramming often involves various epigenetic “writers”, “erasers” and “readers”^55^. To unbiasedly identify potential epigenetic regulators that prevent the development of resistance to Yap inhibition, we carried out a long-term Yap-dependency assay in the presence of vehicle control or various epigenetic inhibitors (Supplementary Table 2). For the initial screen, we selected #5346 cells based on the observations that (1) following Yap KO its proliferation rate declined rapidly but over time recovered to a similar rate as parental cells (Supplementary Fig. 10a), resembling the common course of relapse development in vivo; (2) the restoration in cell proliferation in Yap-KO cells was accompanied by upregulation of PTFs (Supplementary Fig. 10b), again recapitulating what we observed in vivo. For the validation screen, we used human Panc-1 cells, which similar to #5346 showed immediate dependency on YAP and were able to adapt to Yap loss by acquiring PTF expression (Supplementary Figs. 2c, 10c). Strikingly, in both PDAC lines, several BET-i consistently scored among the top hits preventing the outgrowth of Yap-independent populations (Fig. 7a, Supplementary Fig. 10d). To test whether BET-i selectively sensitizes Yap-independent PDAC cells to Yap inhibition, we re-performed short-term Yap dependency assays on all 19 primary PDAC lines in the presence of low-dose BET inhibitor ABBV-075. While ABBV-075 treatment did not enhance the growth inhibitory effects of acute Yap loss on epithelial PDAC cells with low PTF scores, it significantly sensitized intermediate to mesenchymal PDAC cells with high PTF scores to Yap ablation (Fig. 7b).

Yap partners with the TEAD and AP-1 family of TFs to promote PDAC initiation and progression^16,17,56^. Footprint analysis of the Yap Cut&Tag peaks in Yap-dependent #6516 cells confirmed strong binding of Yap to both TEAD and AP-1 sites (Supplementary Fig. 10e). To assess whether BRD4 inhibitor could reduce the development of resistance to YAP/TEAD inhibitor, we conducted in vitro and in vivo combination studies between ABBV-075 and VT103 (a TEAD palmitoylation inhibitor that blocks YAP/TEAD interaction)^24^ using mT3 and mT4 cells, which were derived from the KrasG12D:Trp53R172H:Pdx-Cre (KPC) mice backcrossed into the C57BL/6 background^57^. These two lines exhibited high to intermediate Yap dependency, were able to give rise to Yap-KO-resistant clones expressing various PTFs, and showed selectively sensitivity to ABBV upon genetic silencing of Yap (Supplementary Fig. 10f–h). Treatment of mT3 and mT4 with increasing concentrations of VT103 and ABBV-075 alone or in combination revealed that similar to genetic Yap depletion, VT103, and ABBV-075 strongly synergize in inhibiting the proliferation of these two cell lines in vitro (Fig. 7c). Similarly, combining VT103 and ABBV-075 also significantly further delayed the tumor growth and extended the survival in immune competent C57BL/6 mice bearing mT4 subcutaneous tumors, compared to either treatment alone (Fig. 7d, Supplementary Fig. 11a).

To test whether BET-i treatment could also re-sensitize Yap-independent PDAC cells to YAP/TEAD inhibitors, we treated nude mice carrying #5462 xenografts with vehicle control, VT103, ABBV-075, or ABBV-075 + VT103. As expected, while #5462 tumors treated with VT103 alone grew at a similar rate as vehicle control, they grew considerably slower under treatment with ABBV-075 or ABBV-075 + VT103 (Fig. 7e, Supplementary Fig. 11b). Interestingly, even though the combination treatment initially induced similar delay in tumor growth as ABBV-075 single treatment, it resulted in much more durable tumor control over time (Fig. 7e, Supplementary Fig. 11b), suggesting that ABBV-075 treatment may re-sensitize Yap independent PDAC tumors to YAP/TEAD inhibitors. Mechanistically, we found that ABBV-075 induced the re-sensitization of Yap-independent PDAC cells to genetic or pharmacological inhibition of Yap was accompanied by robust downregulation of PTFs both in vitro and in vivo (Fig. 7f, Supplementary Fig. 11c, d). Finally, ABBV-075 treatment also reduced the levels of PTFs in primary PDAC lines and inhibited the growth of xenografts derived from Yap-KO relapsed lesions (Supplementary Fig. 11e, f).

Taken together, these results suggest that BRD4 is required for promoting the expression of PTFs and BET-i could be used to prevent or overcome resistance to Yap inhibition.

## Discussion

Small molecule inhibitors that block the activity of the master oncogenic transcriptional activator Yap have entered into Phase I clinical trials for the treatment of advanced solid tumors with hyperactivated Yap (NCT04665206; NCT05228015). PDAC, which is not only highly reliant on Yap to maintain their growth and survival but also entails Yap to resist the suppression of Kras or its direct effector pathways, represents a strong candidate for Yap-targeted therapies^15^. Here, we preemptively investigated potential resistance mechanisms to Yap inhibition in advanced PDAC tumors. Using an inducible PDAC GEM model, we showed that similar to the extinction of oncogenic Kras, a significant fraction of advanced PDAC tumors were able to overcome Yap loss and acquire alternative mechanisms to foster their growth. Intriguingly, we found that the relapse lesions tend to arise from the peripherals or near necrotic regions of the tumors, where rare pockets of pre-existing “progenitor”-like PDAC cells tend to reside. These “progenitor”-like express bona fide PTFs including Sox2, Sox5, Twist2, Nr2f1, and/or Nr2f2, but universally lack of expression for the squamous marker deltaNp63, suggesting that they are distinct from PDAC cells that have undergone squamous trans-differentiation. Interestingly, these “progenitor”-like PDAC cells also do not exhibit increased expression in most of the previously reported putative markers of PDAC “cancer stem cells” or “tumor-initiating cells” including Dclk1, Msi2, or Rorc (RORgamma)^58–61^, although they do express elevated levels of pan-CSC markers Cd24a and Cd44.

**Fig. 8:**
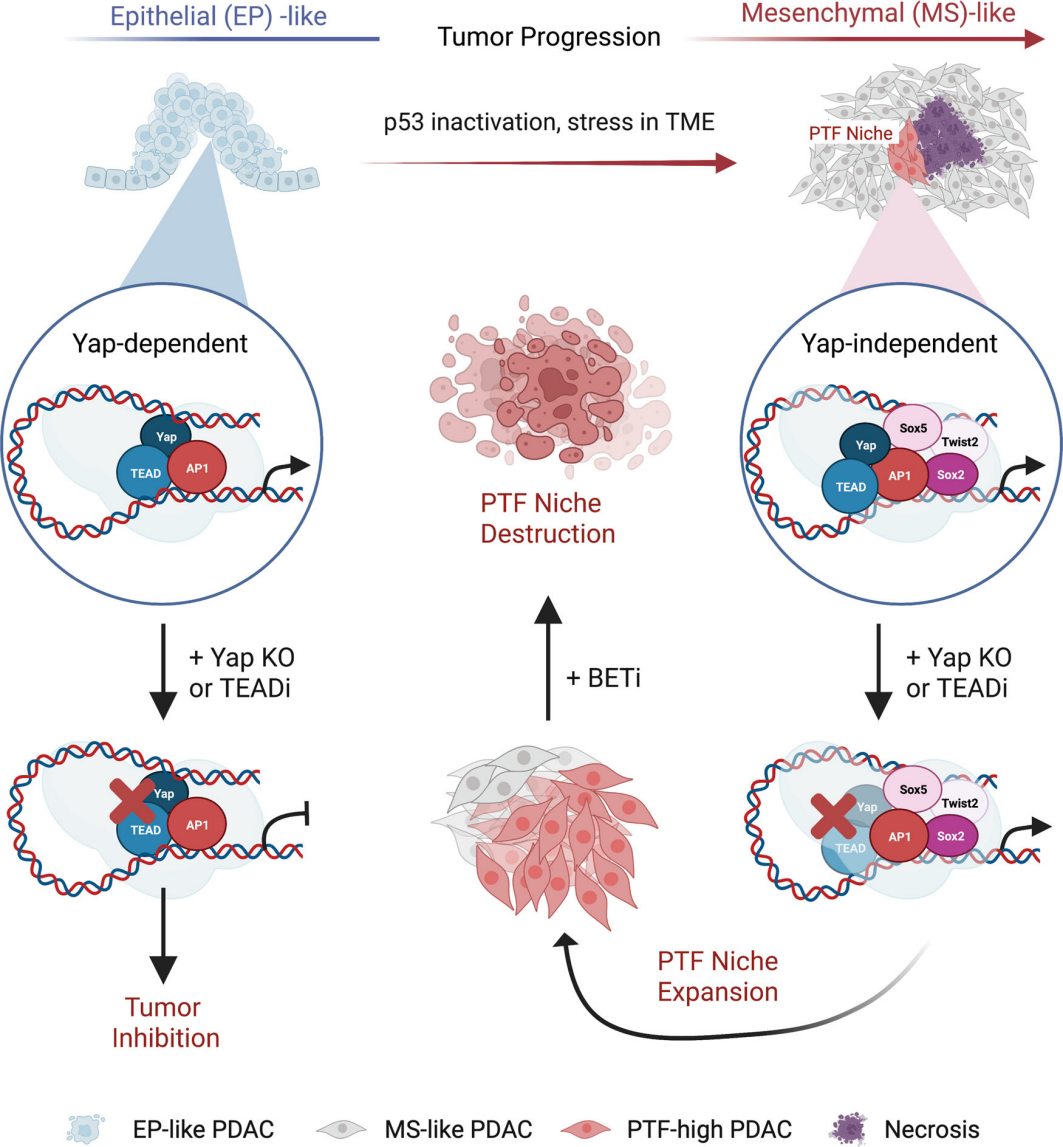
Schematic representation of the current working model based on our findings. Created with BioRender.com.

Our study suggests that adverse TME such as hypoxia, nutrient, or mechanical stress likely provide the instructive cues for the activation of various PTFs (Fig. 8). Even though multiple PTFs tend to co-express within a given “progenitor” niche, each PTF often exhibits partially overlapping but overall distinct distribution patterns from another PTF within the same tumors, suggesting that various PTFs may respond differently to certain environmental cues. Another notable feature of these “progenitor” niches is that they are mostly quiescent in the majority of Yap-expressing advanced PDAC tumors, but become highly proliferative following Yap ablation by re-activating Myc. Future studies will be needed to elucidate how long-term Yap extinction triggers Myc re-activation and outgrowth of these “progenitor”-like cells.

Through comparative analysis of a large panel of primary PDAC cells derived from primary PDAC tumors at various stages of disease progression, we demonstrated that the acquisition of “progenitor”-like states is coupled with the process of EMT (Fig. 8). However, our study also reveals that the “progenitor”-like niches represent only a very small subset of the tumor cells that have undergone morphological EMT and are not activated by commonly used EMT inducer TGFb. Thus, PTFs likely form distinct transcriptional networks from the classic EMT TFs.

Although hundreds of TFs are expressed at any time in a cell, only a small number of master TFs control the core transcriptional programs governing cell identity and survival^62,63^. A growing body of evidence suggests that during tumor progression, tumor cells hijack or activate evolving combinations of interconnected master TFs to maintain their growth and adapt to the complex TME or therapeutic stress^64–75^. These various master regulators form feed-forward autoregulatory loops, and function in a cooperative and partially redundant manner to maintain SEs and the expression of cancer-promoting genes. AP-1 are dimeric transcriptional complexes composed of members of Jun (Jun, JunB, and JunD) and Fos (Fos, FosB, FosL1, FosL2) families. Members of AP-1 proteins including Jun and FosL1 have been shown to be directly regulated by the Ras–MAPK pathway and are required for Ras-induced transformation in vitro and in vivo^56,76–79^. In multiple types of cancer including PDAC, AP-1 is known to cooperate with Yap/Tead to maintain oncogenic transcription^56,80–84^. Our study revealed that Jun also form interconnected feed-forward transcriptional loops with PTFs Sox2/5, Twist2, Nr2f1/2, and recruit PTFs to activate SEs associated with Yap independency. Notably, CREs occupied by individual PTFs or Jun alone are largely devoid of H3K27 acetylation and Brd4 binding, suggesting that the cooperative interactions between PTFs and Jun are required to maintain active transcription in Yap-independent PDAC cells (Fig. 8).

An unexpected discovery from our Cut&Tag profiling of 19 primary PDAC lines was that their relative Yap dependencies are not correlated with the overall binding patterns of Yap. In PTF-high, Yap-independent PDAC cells, the vast majority of Yap-bound sites are co-occupied by three or all four Jun/PTFs, which may explain why they are insensitive to Yap ablation (Fig. 8). The pile-ups of multiple different PTFs at the active TSS/CRE sites including the majority of SE regions may also increase the resiliency of these sites to sudden loss of individual PTFs. Presumably, as long as there are sufficient levels of any of the PTFs remain at these sites, the overall transcriptional homeostasis can be maintained. This scenario fits with our observations that while exogenous expression of a single PTF in PTF-low cells is sufficient to overcome their Yap dependency, silencing of a single PTF is not adequate to cause growth inhibition despite the concomitant partial downregulation of other PTFs.

Through an unbiased chemical-genetic screen, we identified Brd4 as an epigenetic regulator required for maintaining the Jun/PTF CRC, and showed that Yap-independent PDAC tumors with high PTF expression are very sensitive to BET inhibitors (Fig. 8). Moreover, our studies showed that combining BET and Yap/Tead inhibitors resulted in more durable control of PDAC xenografts, highlighting the therapeutic potential of this combination. Our findings are consistent with several independent studies pointing to the unique sensitivities of oncogenic CRCs to BET inhibitors in various cancer types^65,66,74,85^. Given that many cancers are addicted to CRC, a combination of CRC-disrupting agents such as BET inhibitors with either existing treatment modalities or novel antitumor compounds such as Yap/Tead inhibitors may provide much-needed strategies to improve the overall response rates and progression-free survival in cancer patients.

## Methods

### Animal studies

All animal studies were conducted in compliance with ethical regulations according to protocol #2016-1192 approved by the Institutional Animal Care and Use Committee (IACUC) at Georgetown University. All the mice were housed in SPF vivarium which is maintained at a 12:12 h light:dark cycle, at 68–74 °F and 30–70% humidity range. Both female and male mice between the ages of 5–35 weeks old were used in this study. Mice were euthanized at endpoints according to the IACUC guideline with approved methods of CO_2_ followed by cervical dislocation. C57BL/6J wild-type mice (Strain Code: 027) and NCI Athymic NCr-nu/nu mice (Strain Code: 553) were purchased from Charles River Laboratories (Wilmington, MA).

### Genetically engineered mouse model of PDAC

Genetically engineered mouse strains *Yap*^*flox/flox*^, *TP53*^*FRT/+*^, *FSF-Kras*^*G12D*^, *R26*^*CreER*^, *R26*^*Dual*^, and *Pdx1-Flp* were interbred to generate the experimental cohorts^5,32,86^. Both male and female mice were included in the study. TAM-containing diet (TD.130859; Envigo, Somerset, NJ) and Dox-containing Diet (TD0.1306; Envigo) purchased from Envigo RMS Inc, Indianapolis, IN was given in place of regular feed.

### Xenograft studies

5 × 10^4^ PDAC cells were injected into the flanks of 7-week-old male NCr-nu/nu mice (#5462 cells and #6385 cells) or C57BL/6J (mT4 cells). Tumor volume was measured with a caliper every other day, and Dox or drug treatment was initiated once the tumor volume reached 100 mm^3^. For inhibitor studies, DMSO, 0.5 mg/kg ABBV-075 (ABBV), 30 mg/kg VT103, or a combination of ABBV and V103 (*n* = 6 mice each) were administered daily via oral gavage. The mice were euthanized at endpoints according to the IACUC guideline. Tumor volume was calculated according to the formula Volume (mm^3^) = Length (mm) × Width (mm)^2^/2, with the maximum tumor size of 2000 mm^3^ as required by IACUC. Statistical analysis was performed using The GraphPad Prism software. Kaplan-Meier survival curve was used to estimate the difference in lifespan between experimental arms. 2-way repeated measure ANOVA was used to determine the significant difference (if any) in growth rates between the experimental arms. Significance is defined as a *p*-value of <0.05. Error bars on all graphs indicate standard errors from the mean.

### Isolation of primary PDAC cells

The tumor-bearing pancreata from KPF and KPYYF mice were divided into three parts for collecting tumor cells, fixing in 10% buffered formalin (for IHC/IF), and snap-frozen in liquid nitrogen for RNA and protein extractions. The fresh tumor was digested with 0.32 mg/mL Collagenase D (Sigma) and 0.01 mg/mL DNaseI (Sigma) in DMEM for 30 min at 37 °C on a shaker and passed digested tissue through a 40 μm cell strainer. The remaining undigested tissue was collected, washed with PBS, further digested with 0.25% Trypsin (Fisher Scientific) for 10 min at 37 °C, and passed through a 40 μm cell strainer. GFP+ or Tm+ tumor cells were collected by FACS sorter (BD Biosciences, Franklin Lakes, NJ), plated in 10% FBS medium for establishing primary PDAC lines, or saved at −80 °C for RNA extraction.

### Cell lines

Panc1 (CRL-1469) and 293T (CRL-3216) cells were purchased from ATCC. mT3 and mT4 mouse PDAC cell lines were provided by Dr. Tuveson (Cold Spring Harbor Laboratory, Cold Spring Harbor, NY) and authenticated by genotyping PCR to compare with Kras^+/LSL-G12D^; Trp53^+/LSL-R172H^; Pdx1-Cre (KPC) model^87^. Established primary PDAC cell lines were authenticated by genotyping PCR to compare with the corresponding mice. Lentiviral plasmids pTRIPZ, TLCV2, and pCW57 were purchased from Addgene. Specific shRNA and sgRNA sequences were listed in Supplementary Table 4. Gateway Entry plasmids for SOX2 (HsCD00436328), SOX5 (HsCD00442638), TWIST2 (HsCD00330331), and NR2F2 (HsCD00005215) were purchased from DNASU (Tempe, AZ), and transferred into the pCW57 destination vector using Gateway LR Clonase II Plus (Thermo Fisher Scientific, Waltham, MA) according to the manufacturer’s instructions. Lentiviral production was performed as previously described^17^. For pTRIPZ or TLCV2-infected PDAC cells, puromycin selection was used to eliminate uninfected cells. For pCW57-infected PDAC cells, FACS was used to purify PDAC cells carrying overexpressing vectors. Cells were grown in DMEM supplemented with 10% Fetal Bovine Serum and 1% Pen/Strip (Thermo Fisher Scientific). All cell lines were free of mycoplasma infection using PlasmoTest Mycoplasma Detection Kit (InvivoGen, San Diego, CA).

### RNA sequencing

Total mRNAs were extracted from FACS-sorted GFP+ or Tm+ PDAC cells using an RNAeasy Mini kit (Qiagen, Hilden, Germany) according to the manufacturer’s instructions. Reverse transcription, library preparation, and hybridization to Illumina MouseRef-8 v2.0 Expression BeadChips were performed by the UCLA Neuroscience Genomics Core. Raw intensity data were background corrected and filtered of low expression genes across samples (∑ < 100 read) prior to analysis. Normalization and differential expression were performed in R using the DESeq2 package from Bioconductor. A cutoff of *p* < 0.05 and |FC| > 2 were used to determine differentially expressed genes. The GSEA Java-based software package from The Broad Institute was used to identify top enriched pathways in Tm+Yap− cells relative to GFP+Yap+ cells.

### Western blotting

Frozen tumor chunks were grounded with a mortar and pestle chilled by liquid nitrogen. Powdered tissues or cell pellets were lysed with Urea buffer (9.5 M urea, 2% CHAPS, 0.5% sodium deoxycholate) and subjected to SDS–PAGE and western blot as previously described^20^. All primary and secondary antibodies used are listed in Supplementary Table 4. All uncropped and unprocessed scans of the blots presented in the figures can be found in the Source Data file or as a supplementary figure in the Supplementary Information.

### IHC/IF

All antibodies used for IHC and IF were listed in Supplementary Table 4.

For IHC, formalin-fixed and paraffin-embedded tumor sections were deparaffinized and heated in antigen retrieval buffer as indicated (IHC-Tek^TM^ Epitope Retrieval Solution, IHC World LLC, Woodstock, MD; or 10 mM Tris Base, 1 mM EDTA Solution, 0.05% Tween 20, pH 9.0) for 30 min at 95 °C. After the slides cool down to RT, they were washed 2 times with PBST (PBS + 0.1% Tween-20), blocked with 3% H_2_O_2_ for 15 min, then with 5% normal horse serum for 30 min, followed by incubation overnight with primary antibodies at 4 °C. Next day, slides were washed for 6 times with PBST, incubated with corresponding IMMPRESS HRP POLYMER REAGENTS (Vector Laboratories, Burlingame, CA) for 1 h at RT. After washing with PBST, staining was visualized using the ImmPACT DAB EqV HRP substrate (Vector Laboratories) according to the manufacturer’s instructions. The entire IHC slides were scanned using the Hamamatsu NanoZoomer slide scanning system (the Alafi Neuroimaging Laboratory, St. Louis, MO) and images were analyzed using NDP.view2 Viewing software (Hamamatsu Photonics, Shizuoka, Japan) and FIJI. Tumor area was determined by the total area of GFP+ and Tm+ staining. The recombination ratio was calculated by Tm+ region within the tumor region.

IF of primary PDAC cells was performed as previously described using fluorescein-conjugated secondary antibodies^20^. Confocal fluorescence images were obtained with the Leica SP8 confocal microscope and processed using FIJI.

### Determining Yap dependency scores

GFP+Yap+ and Tm+Yap− derived from the same primary murine PDAC lines were mixed in equal proportions and grew in co-cultures. The relative percentages of the two populations were tracked over time through sequential FC analysis. The Yap dependency scores were calculated by percentages of GFP+ and Tm+ populations following Dox treatment. Each experiment was performed in triplicates and Student’s *t*-test was used to determine the difference in Yap dependency scores between the samples. Significance is defined as a *p*-value of 0.05 or less. Error bars on all graphs indicate standard errors from the mean.

### Epigenetic inhibitor screening and drug synergy studies

All inhibitors used are listed in Supplementary Table 3. For epigenetic inhibitor screening, GFP+Yap+ and Tm+Yap− #5346 primary murine PDAC cells, or ctrl and shRNA-targeting YAP introduced human Panc1 cells were mixed in equal proportions and split into 24-well plates with each well containing DMSO control or a different epigenetic inhibitor. The inhibitor-containing medium was replaced every three days and sequential FC analysis was performed weekly for a month. mT3 or mT4 PDAC cells were treated with DMSO control or increasing concentrations of BET and TEAD inhibitors alone or in combination. Once the DMSO control wells each confluence, the plates were fixed in 4% paraformaldehyde, stained with crystal violet, and measured by Synergy™ H4 Hybrid Multi-Mode Microplate Reader (BioTek, Winooski, VT). SynergyFinder (https://synergyfinder.fimm.fi/) was used to calculate the synergy scores based on the percent of inhibition relative to DMSO.

### Cut&Tag

All antibodies used for Cut&Tag were listed in Supplementary Table 4. Cut&Tag was carried out according to the bench top Cut&Tag V.3 with minor modifications^88^. Primary PDAC cells were harvested using a cell stripper (Thermo Fishers), counted and centrifuged for 3 min at 800 × *g* at room temperature. Aliquots of 250,000 cells per antibody were washed twice in 100 µL per sample of Wash Buffer (20 mM HEPES pH 7.5, 150 mM NaCl, 0.5 mM Spermidine, 1× Protease inhibitor cocktail) by gentle pipetting. Concanavalin A (ConA) coated magnetic beads (Bangs Laboratories, Fishers, IN) were washed twice with ConA Binding Buffer (20 mM HEPES pH7.5, 10 mM KCl, 1 mM CaCl_2_, 1 mM MnCl_2_) and 10 µL of activated beads were added per sample and incubated at RT for 15 min on a rotator. ConA bead-bound cells were placed on the magnetic stand and the unbound supernatant was removed. ConA bead-bound cells were resuspended in ice-cold 100 µL Dig-wash Buffer (20 mM HEPES pH 7.5, 150 mM NaCl; 0.5 mM Spermidine, 1× Protease inhibitor cocktail, 0.05% Digitonin) containing 2 mM EDTA and diluted primary antibody indicated on Supplementary Table 4. Primary antibody incubation was performed on a rotating platform for 2 h at room temperature (RT). The primary antibody was removed by placing the tube on the magnet stand to clear and pulling off all of the liquid. Guinea Pig anti-Rabbit IgG secondary antibody was diluted 1:100 in 100 µL of Dig-Wash buffer and cells were incubated at RT for 1 h. Cells were washed using the magnet stand 5 times in 150 µL Dig-Wash buffer to remove unbound antibodies. After removing the liquid on the magnet stand, 100 µL protein A (pA)-Tn5 adapter complex diluted 1:100 in Dig-300 Buffer (0.05% Digitonin, 20 mM HEPES, pH 7.5, 300 mM NaCl, 0.5 mM Spermidine, 1× Protease inhibitor cocktail) was added to the cells with gentle vortexing and incubated at RT for 1 hr. pA-Tn5 adapter complex was prepared by mixing pA-Tn5 (Addgene) fusion protein with preannealed mosaic end-adapter A and -adapter B and incubation for 1 h at RT. Cells were washed 5 times in 150 µL Dig-300 buffer to remove unbound pA-Tn5 protein. Cells were resuspended in 200 µL Tagmentation buffer (10 mM MgCl_2_ in Dig-300 Buffer) and incubated at 37 °C for 1 h. To stop tagmentation, 6.67 µL of 0.5 M EDTA, 2 µL of 10% SDS and 1.67 µL of 20 mg/mL Proteinase K was added to the sample, and incubated at 50 °C for 30 min, and then at 37 °C overnight. To extract the DNA, 200 µL PCI was added and mixed with vortexing. 200 µL vacuum grease was added to separate layers of protein and DNA, and tubes were centrifuged for 3 min at RT 16,000×*g*. 200 µL Chloroform was added and inverted 10 times, and tubes were centrifuged for 3 min at RT 16,000 × *g*. The aqueous layer was transferred to a new tube containing 500 µL ice-cold 100% ethanol and centrifuged for 15 min at 4 °C 16,000 × *g*. The pellet was rinsed with 100% ethanol and centrifuged for 2 min at 4 °C 16,000 × g. After the liquid was aspirated, the pellet was dissolved in 30 µL 10 mM Tris–HCl pH 8, 1 mM EDTA containing RNaseA. PCR was performed with NEBNext HiFi 2x PCR Master mix (NEB, Ipswich, MA), unique i7 barcode primer, and Universal i5 primer. To extract the DNA, 0.4 volume Mag-Bind® TotalPure NGS beads (Omega Bio-Tech, Norcross, GA) were added to each tube with vortexing, quickly spun, and held for 10 min. Tubes were placed on a magnet stand to clear, then the liquid was transferred to a new tube. 0.7 volume Mag-Bind® TotalPure NGS beads were added to each tube with vortexing, quickly spun, and held for 10 min. Tubes were placed on a magnet stand to clear, then the liquid was carefully withdrawn. Without disturbing the beads, beads were washed twice in 200 µL 80% ethanol. After allowing to dry ~5 min, 30 µL of 10 mM Tris pH 8 was added, the tubes were vortexed, quickly spun, and allowed to sit for 5 min at 37 °C. Tubes were placed on a magnet stand and the liquid was withdrawn to a fresh tube.

### Quantification and sequencing of Cut&Tag libraries

Barcoded Cut&Tag libraries were prepared using NEBNext HiFi 2x PCR Master mix (NEB) with Nextera i7 and i5 dual index primers, purified with Mag-Bind® TotalPure NGS beads, quantified via qPCR using the KAPA Library Quantification Kit (Roche, Basel, Switzerland). Pooled libraries were sequenced on the Illumina High-seq platform (Read 1:150 cycles, Index 1:8 cycles, Index 2:8 cycles, Read 2:150 cycles).

### Pre-processing of Cut&Tag sequencing data

Raw sequencing reads from fastq files were trimmed using TrimGalore^89^. Reads were aligned sequentially to bacteria and the mm10 genome using Bowtie2^90^ with options -very-sensitive-local -no-mixed -dovetail -phred33 -X 1000 -interleaved. Duplicates were removed using Picard tools (https://github.com/broadinstitute/picard.git), and the blacklisted regions were filtered out using BEDtools to generate filtered BAM files^91^. Tag directories were generated from filtered BAM files using the HOMER makeTagDirectory command with the “-sspe” option^92^. Normalization factors for all the samples were calculated using the “csaw” R package with the settings of minq = 20, max.frag = 800, pe = both, bin = TRUE, width = 1250 for TFs, or minq = 20, max.frag = 800, pe = both, bin = TRUE, width = 10,800 for histones or BRD4^93^. Normalized bigwig files were created with the HOMER makeUCSCfile command with the -bigwig option and the normalization factor calculated by csaw applied using -norm. Peaks were called on individual samples using the HOMER findPeaks command with the options -style factor -L 15 -localSize 150,000 -fdr 0.00001, and normalized by applying the normalization factor calculated by csaw via the “-norm” option.

### PCA and unsupervised clustering analysis

Filtered BAM files for each antibody were computed to assess the genome-wide similarities among the primary PDAC lines using the multiBamSummary bins command from deepTools v2.0 with the “–extendReads” option^94^. The resultant read coverage matrixes were then used to perform PCA and unsupervised clustering analysis using the deepTools plotPCA and plotCorrelation (–corMethod spearman) commands, respectively. The correlation matrixes generated by plotCorrelation were then used to plot heatmps using the “pheatmap” R package with the following settings: scale = “none”, cluster_cols = TRUE, cluster_rows = TRUE, clustering_distance_cols = “euclidean”, clustering_distance_rows = “euclidean”, clustering_method = “ward.D2”.

### Identification of enhancers and promoters associated with Yap dependency

The HOMER makeTagDirectory command with the “-d” option was sued to merge Tag directories from individual samples to generate a single master Tag directory for each antibody. Master peaks were called from the master Tag directory using HOMER findPeaks command with the options -style factor -L 15 -localSize 150,000 -fdr 0.00001, and filtered to remove peaks that did not overlap with any of the peaks detected in individual samples using the bedtools intersect -u command. A master table was then generated for each antibody using the HOMER annotatePeaks.pl command with the options of -size given -raw to extract Tag densities from individual Tag directories overlapping the master peaks normalized with the csaw normalization factors from above using the “DGEList” and “cpm” functions from “edgeR”.

To identify enhancers and promoters associated with Yap dependency, H3K27ac, and BRD4 master peaks were further filtered to remove those detected in fewer than 3 samples, or with maximal normalized Tag counts <10 among all samples, or did not overlap with the master peaks of the other antibody. The resultant set of overlapping H3K27ac and BRD4 master peaks were each subjected to Kendall correlations comparing the normalized Tag counts to the corresponding Yap dependency scores across all the samples. The “RRHO” r packages were used to plot the corresponding correlation efficiencies from the overlapping H3K27ac and BRD4 master peaks. The H3K27ac and BRD4 master peaks with Kendall correlation co-efficiencies both >0.3 or <−0.3 were deemed as concordantly regulated. Yap master peaks overlapping with the concordantly regulated H3K27ac and BRD4 master peaks were extracted using the bedtools intersect function. Log2-transformed normalized H3K27ac, BRD4, and Yap Tag counts of all primary PDAC lines corresponding to the concordantly regulated H3K27ac and BRD4 master peaks were subjected to unsupervised clustering using the “pheatmap” R package with the following settings: scale = “row”, cluster_cols = TRUE, cluster_rows = TRUE, clustering_distance_cols = “euclidean”, clustering_distance_rows = “euclidean”, clustering_method = “ward.D2”.

### Motif and gene set enrichment analysis

To predict TF motifs or biological functions/pathways or positively or negatively associated with Yap dependency, the concordantly regulated BRD4 master peaks from above were subjected to differential motif enrichment analysis using the HOMER findMotifsGenome.pl command with the option of -size 300, and GREAT analysis with default settings. The top 10 most differentially enriched motifs based on p-values were plotted^47^. GeneSetClustering (https://github.com/TranslationalBioinformaticsUnit) was performed using combined GREAT data set^95^. 11 clusters were determined by the silhouette analysis on Kmeans clustering.

### Comparisons of binding profiles of Jun and PTFs

The aggregated signal profiles from Sox2, Sox5, Twist2, and Jun Bigwig files with ±2 kb from the center of BRD4 peaks associated with high or low Yap dependency were plotted using the computeMatrix reference-point and plotProfile commands from deepTools.

For plotting heatmap and assessing the binding patterns, H3K27ac, H3K4me3, BRD4, Sox2, Sox5, Twist2, and Jun peaks were merged to generate a master bed file using the bedtools merge command. All individual bed files were subsequently intersected with the master bed file using the bedtools intersect -wa -wb command. For plotting heatmap, the master peaks were divided into seven peak clusters: p1 (H3K4me3+H3K27ac+BRD4+cJun+Sox2+), p2 (H3K4me3+H3K27ac+BRD4+cJun-Sox2+), p3 (H3K4me3+& not in p1-2), e2 (H3K4me3-H3K27ac-BRD4-cJun+Sox2+), e3 (H3K4me3-H3K27ac-BRD4-cJun+Sox2−), e4 (H3K4me3-H3K27ac-BRD4-cJun-Sox2+), e1 (H3K4me3- & not in e2-4). The computeMatrix reference-point and plotHeatmap commands from deepTools were used to plot the signals from individual Bigwig files within ±3 kb from the centers of seven peak clusters from above. For comparing overlaps among the antibodies, the master peaks were separated into four clusters: active CRE (H3K4me3-H3K27ac+BRD4+), inactive CRE (H3K4me3-H3K27ac|BRD4−), active TSS (H3K4me3+H3K27ac+BRD4+), inactive TSS (H3K4me3+H3K27ac|BRD4−). The numbers of active or inactive CRE/TSS bound by none or various combinations of PTFs and Jun were plotted. Alternatively, the ROSE algorithm^96,97^ was utilized to identify SE regions using H3K27ac or BRD4 Cut&Tag data under default settings. Next, master peaks overlapping the SE or RE regions were extracted. The numbers of master peaks overlapping the SE or RE regions bound by none or various combinations of PTFs and Jun were plotted.

### TF footprint analysis

For the detection of TF footprints, we analyzed BAM files from individual TFs using the TOBIAS package under default settings^52^. The “JASPAR2022_CORE_vertebrates_non-redundant_pfms_jaspar.txt” file was downloaded from “https://jaspar.genereg.net/downloads/”. To enable comparisons among the antibody–motif pairs, we defined “flanking accessibility” or “flank height” as the aggregated signals of the regions flanking a given set of footprints relative to the local background, “footprint depth” as the signal differences between the flanking regions and the core sequences of the footprints, and “relative footprint depth” as “footprint depth”/“flank height”. We defined peaks containing matching motifs regardless of footprint statuses as “all”, among which those with detectable footprints were deemed as “bound”, and those without detectable footprints as “unbound”.

### Reporting summary

Further information on research design is available in the Nature Portfolio Reporting Summary linked to this article.

## Supplementary information

Supplementary Information

Description of Additional Supplementary Files

Supplementary Software 1

Reporting Summary

## Data Availability

The raw fastq data generated from RNA and Cut&Tag sequencing performed in this study were deposited in the GEO database under accession codes GSE224566 and GSE210412, respectively. The reference series is deposited under GSE224567. The Clinical Proteomic Tumor Analysis Consortium (CPTAC) publicly available data used in this study are available in the LinkedOmics under pancreatic adenocarcinoma (http://www.linkedomics.org/data_download/CPTAC-PDAC/)^37^. The Cancer Genome Atlas (TCGA) publicly available data used in this study are available in the GDC Data Portal under TCGA-PAAD (https://portal.gdc.cancer.gov/projects/TCGA-PAAD)^38^. The remaining data are available within the Article, Supplementary Information, or Source Data file. Source data are provided as a Source Data file. Source data are provided with this paper.

## Source data

Source Data

## Code availability

All the source code and software used for the analysis performed for this study are available in Supplementary Software 1.
