# Supplementary material for: Spontaneously evolved progenitor niches escape Yap oncogene addiction in advanced pancreatic ductal adenocarcinomas
Source: Nat Commun. 2023 Mar 15;14:1443. doi: 10.1038/s41467-023-37147-y (PMC10017707; doi:10.1038/s41467-023-37147-y)
Supplement: Supplementary file 1 — Supplementary Information [file 41467_2023_37147_MOESM1_ESM.pdf]

Supplementary Information

Spontaneously Evolved Progenitor Niches Escape Yap Oncogene Addiction in Advanced Pancreatic Ductal Adenocarcinomas

Shigekazu Murakami, Shannon M White, Alec T McIntosh, Chan D.K. Nguyen, and Chunling Yi

Supplementary Figure 1

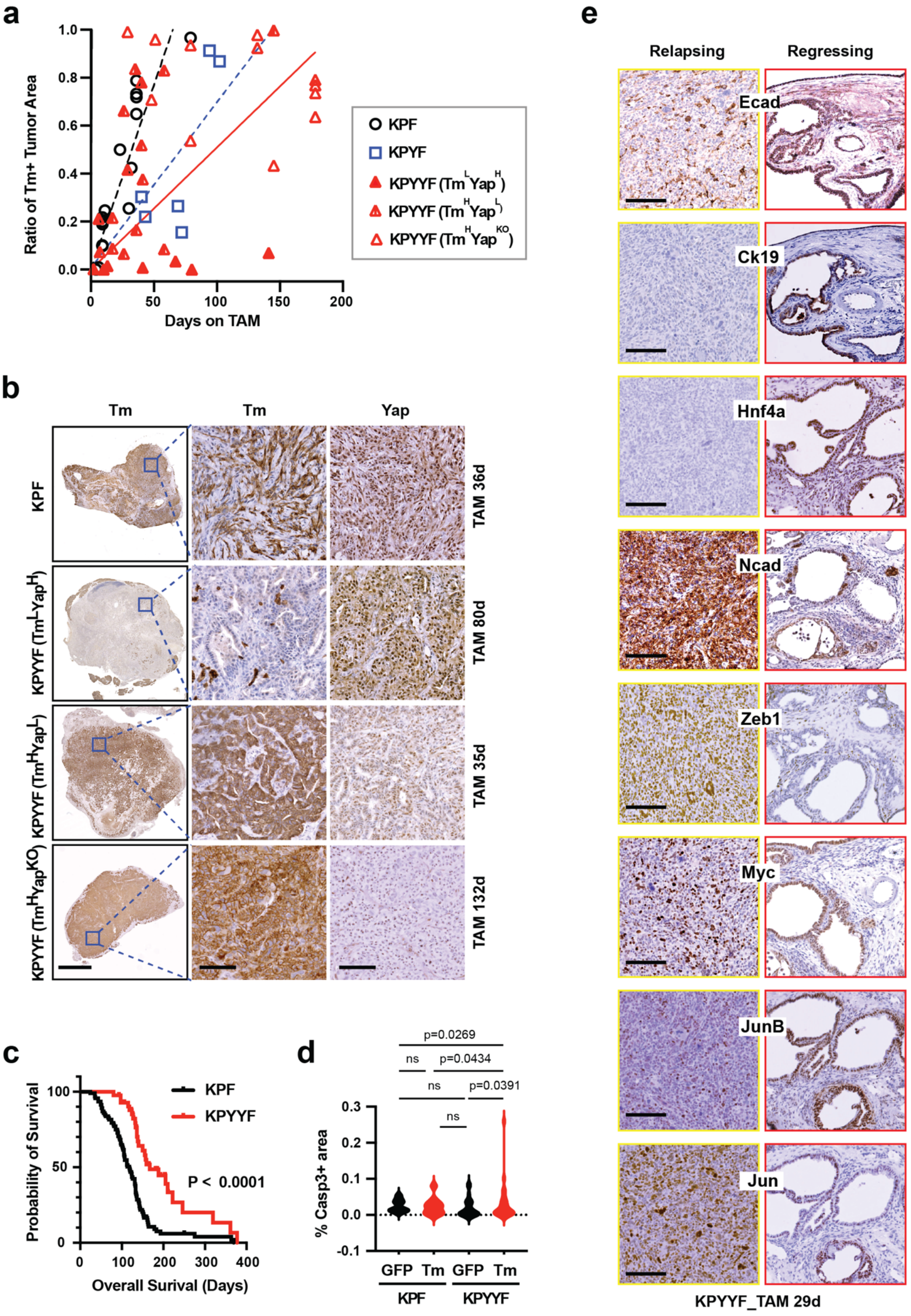

Supplementary Figure 1. TAM treatment prolongs the survival of tumor-bearing mice despite divergent rates of Yap ablation. a. Scatter plot showing the tumor recombination rates calculated by dividing Tm+ area over Tm+ and GFP+ area and the corresponding days of TAM treatment of individual KPF, KPYF and KPYYF mice. Symbols represent genotypes whether KPF (circle), KPYF (square), or KPYYF (triangle). KPYY tumors are further grouped into three classes: $Tm^{L}Yap^{H}$ (low percentages of Tm+ cells and high Yap expression; filled triangle), $Tm^{H}Yap^{L}$ (high percentages of Tm+ cells with low Yap expression; half-filled triangle), and $Tm^{H}Yap^{KO}$ (high percentages of Tm+ cells with complete knockout Yap; open triangle). Trendlines indicate the average changes in recombination rates over time among KPF (black dash), KPYF (blue dash), and all of the KPYYF mice (red lines), respectively. **b.** Representative IHC images for Yap and Tm in PDAC tumors from KPF and the three major types of KPYYF mice. The days of TAM treatment for individual mice were shown at the bottom of images. Scale bars indicate 250 mm (left) or 100 μm (middle, right). Experiments were performed on KPF (n=9), KPYYF ($Tm^{L}Yap^{H}$;n=6), KPYYF ($Tm^{H}Yap^{L}$;n=11), and KPYYF ($Tm^{H}Yap^{KO}$;n=7). **c.** Kaplan-Meier survival curve from KPF (n = 42) and KPYYF (n = 92) mice. Mantel-Cox Long Rank test. **d.** Percentage of CC3+ area within GFP+ or Tm+ areas in KPF (n = 9) and KPYYF ($Tm^{H}Yap^{L}$ and $Tm^{H}Yap^{KO}$; n = 15) tumors. Unpaired, one-tailed, Student's t-test. **e.** Representative IHC images of indicated antibodies at relapsing (yellow) and regressing (red) regions of a KPYYF PDAC tumor after 29 days of TAM treatment matching Figure 1c. Scale bars indicate 50 μm. Experiments were performed on at 13 different KPYY mice. Source data are provided as a Source Data file.

Supplementary Figure 2

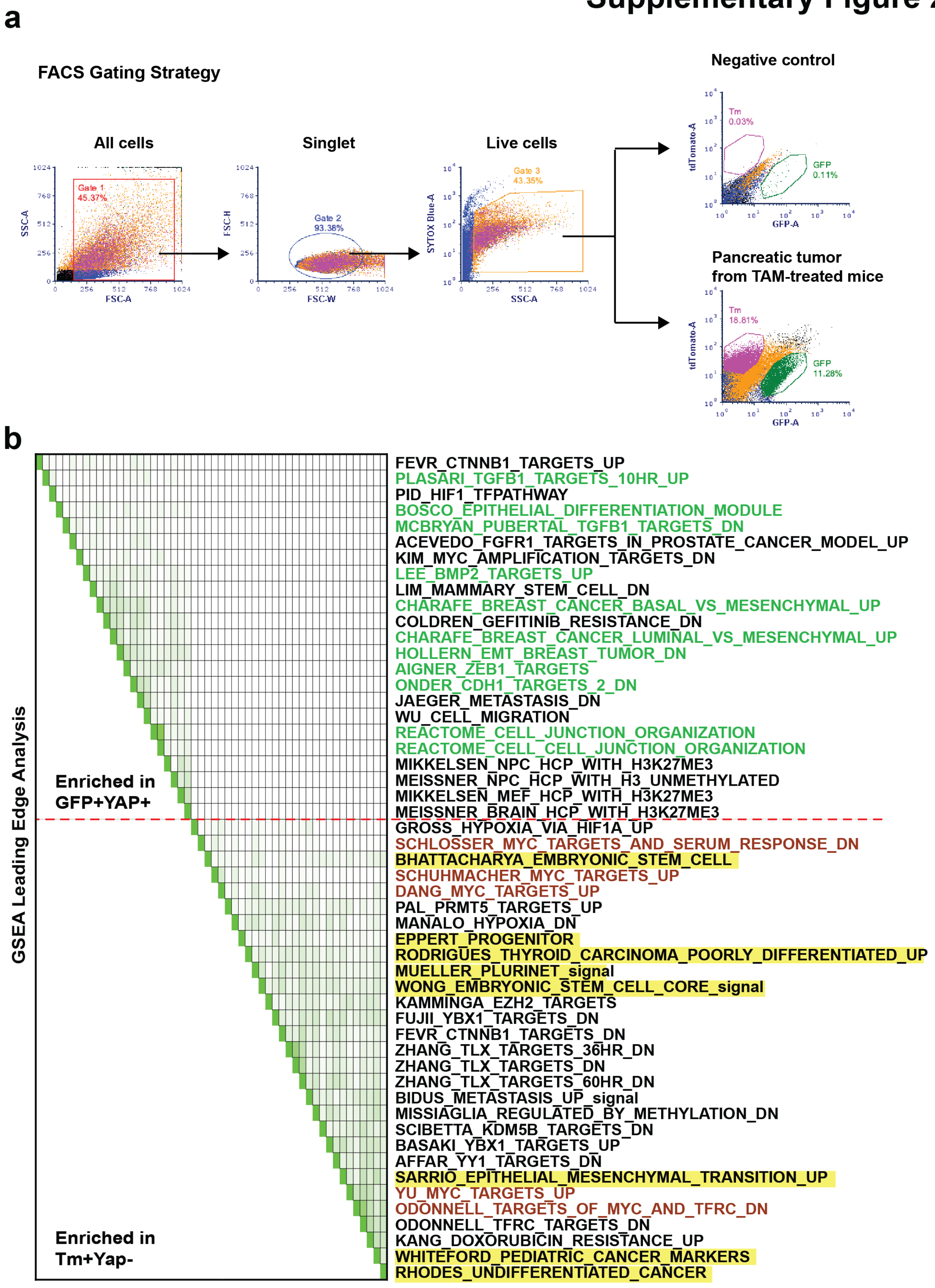

Supplementary Figure 2

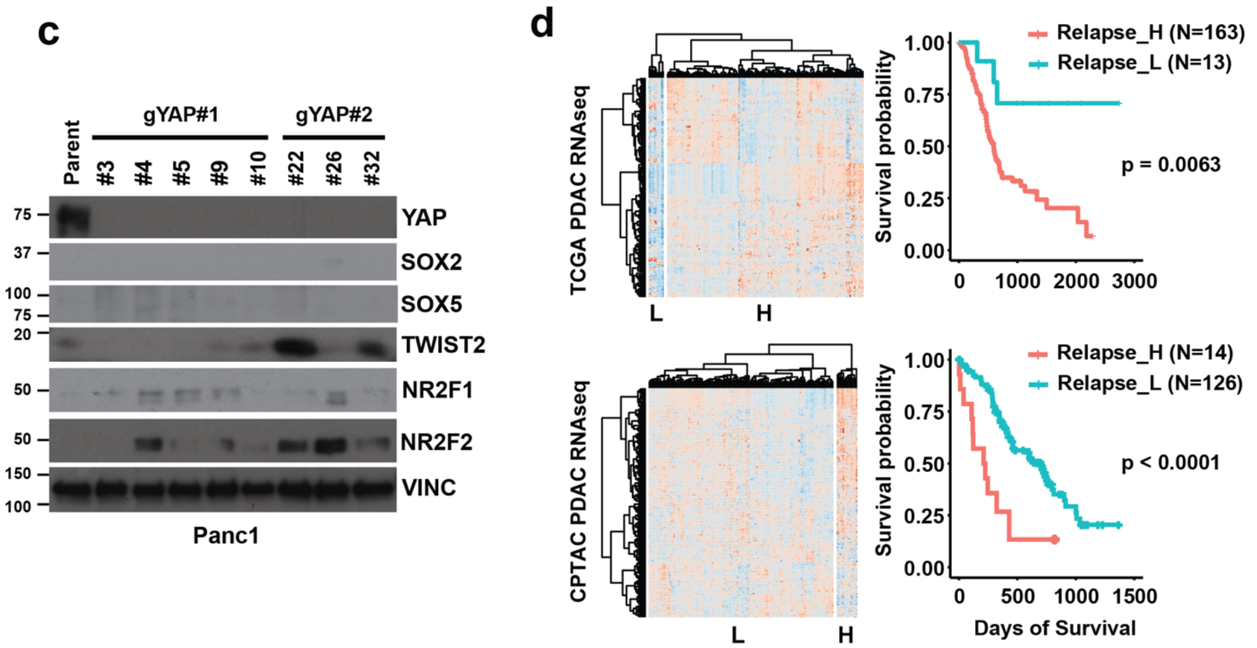

Supplementary Figure 2. The gene signature upregulated in relapsed KPYYF tumors is correlated with worse survival in human PDAC patients. a. Gating strategy for fluorescence-activated cell sorting (FACS) of GFP+ or Tm+ PDAC cells from the pancreata of KPF or KPYYF mice. **b.** Leading-edge analysis of the top-enriched Gene Sets in Tm+/Yap- or GFP+/Yap+ PDAC cells according to GSEA from Figure 1d. Gene Sets related to epithelial differentiation and cell-cell junctions are colored in green; Gene Sets related to Myc targets are colored in brown; Gene Sets related to pluripotent/progenitor and embryonic stem cells, undifferentiation and EMT are highlighted in yellow. **c.** WB analysis of indicated proteins in parental human Panc1 cells or YAP CRISPR knockout clones generated using two different YAP gRNAs (gYAP #1 and #2). VINC was used as the loading control. Shown is representative of three independent experiments. **d.** Left: Heatmaps depicting results from unsupervised clustering of TCGA (top; n = 176) and CPTAC (bottom: n = 140) PDAC tumors based on the mRNA expression of Yap-KO relapse-associated genes identified by RNA-seq of Tm+/Yap- and

GFP+/Yap+ murine PDAC cells from Figure 1d (right). Right: Kaplan Meier survival analysis of TCGA (top) and CPTAC (bottom) PDAC patients expressing high (H) or low (L) levels of Yap-KO relapse-associated genes. TCGA (top): Relapse_H (n=163) and Relapse_L (n=13), and CPTAC (bottom): Relapse_H (n=14) and Relapse_L (n=126), respectively. Mantel-Cox Long Rank test. Source data are provided as a Source Data file.

Supplementary Figure S3

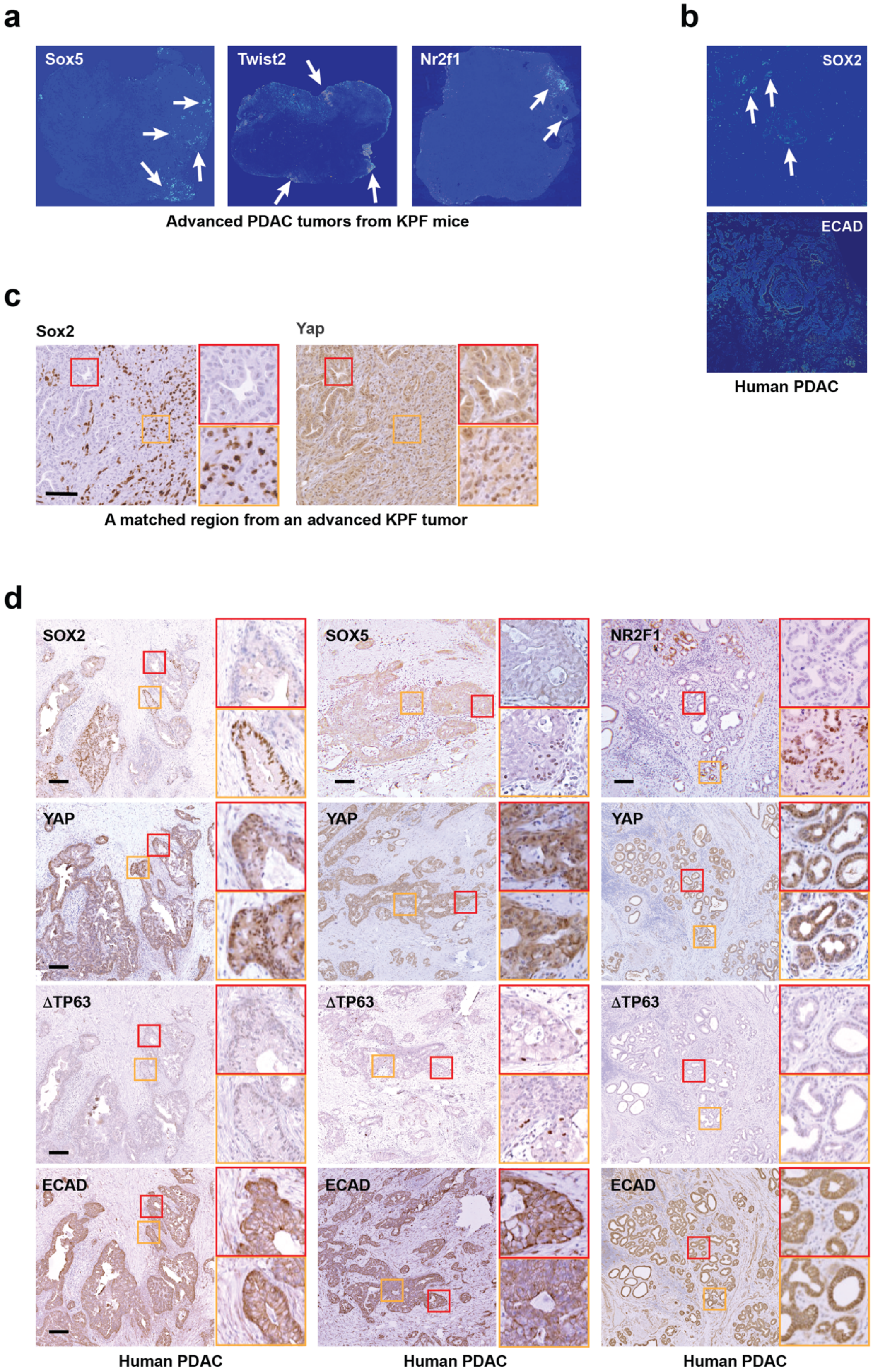

Supplementary Figure 3. PTF-expressing progenitor niches are present in both advanced murine and human PDAC tumors. a. Heatmaps of representative IHC staining of Sox5, Twist2, and Nr2f1 in three different advanced PDAC tumors from KPF mice. **b.** Heatmaps of representative IHC staining of SOX2 and ECAD at a matched region of an invasive PDAC tumor from a human patient. **c.** IHC images of Sox2 and Yap at a matched region of advanced PDAC tumor from a KPF mouse with two boxes marking two representative Sox2+ (yellow) and Sox2- (red) subregions. Scale bar indicates 200 µm. IHC staining for the same set of antibodies was performed on 16 different mice. **d.** IHC images of SOX2, SOX5 and NR2F1 with matching images of YAP, ΔTP63 and ECAD at different regions of an invasive PDAC tumor from a human patient. IHC staining for the same set of antibodies was performed on five human PDAC tumor samples. Scale bar indicates 100 µm.

Supplementary Figure 4

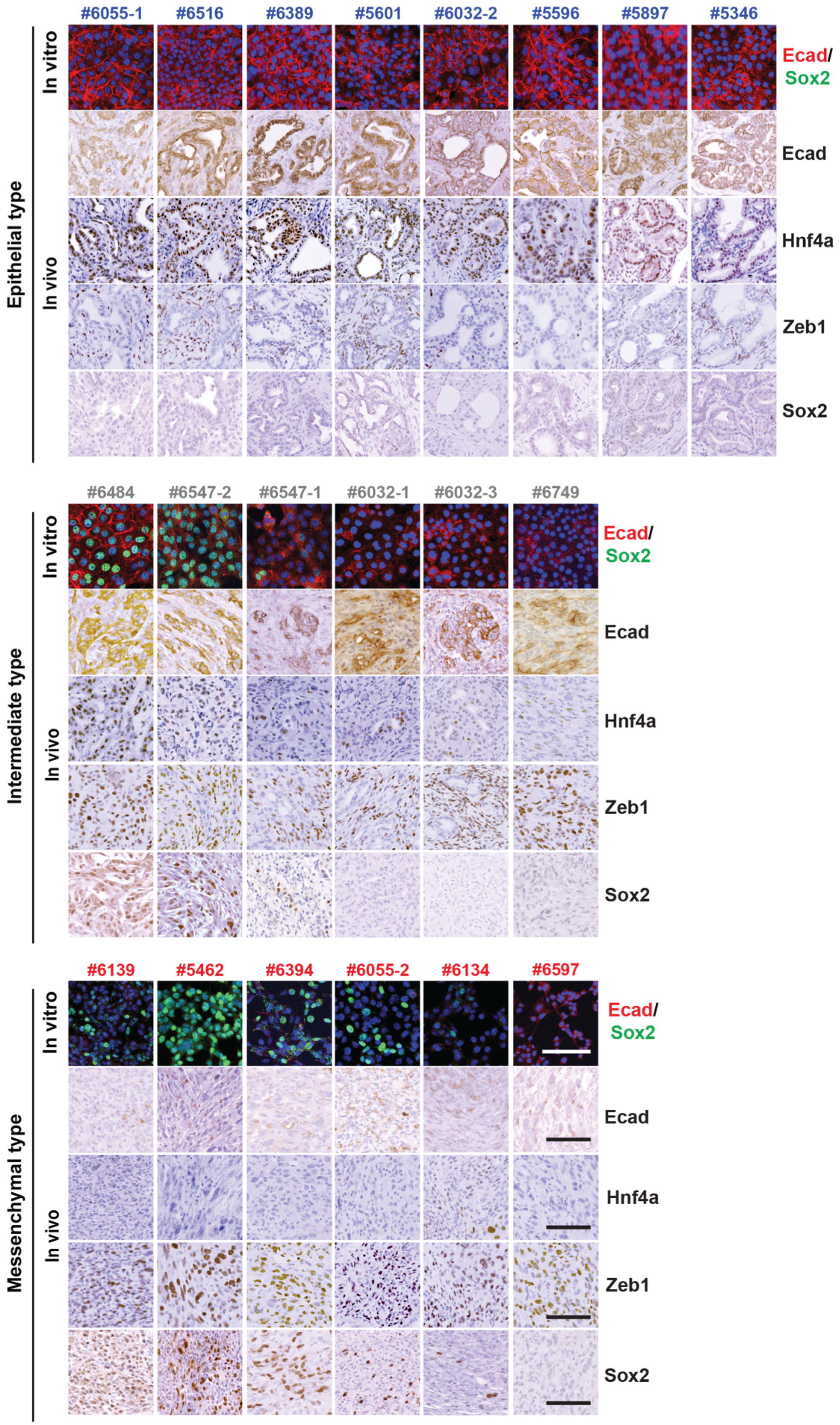

Supplementary Figure 4. PTF expression is associated with EMT. Representative images from IF (top panel) analysis of primary PDAC cell lines against Ecad (red) and Sox2 (green) and from IHC analysis of the corresponding primary PDAC tumors with indicated antibodies. N= 8, 6 and 6 mice for Epithelial, Intermediate, and Mesenchymal groups, respectively. Scale bars indicate 100 μm.

Supplementary Figure 5

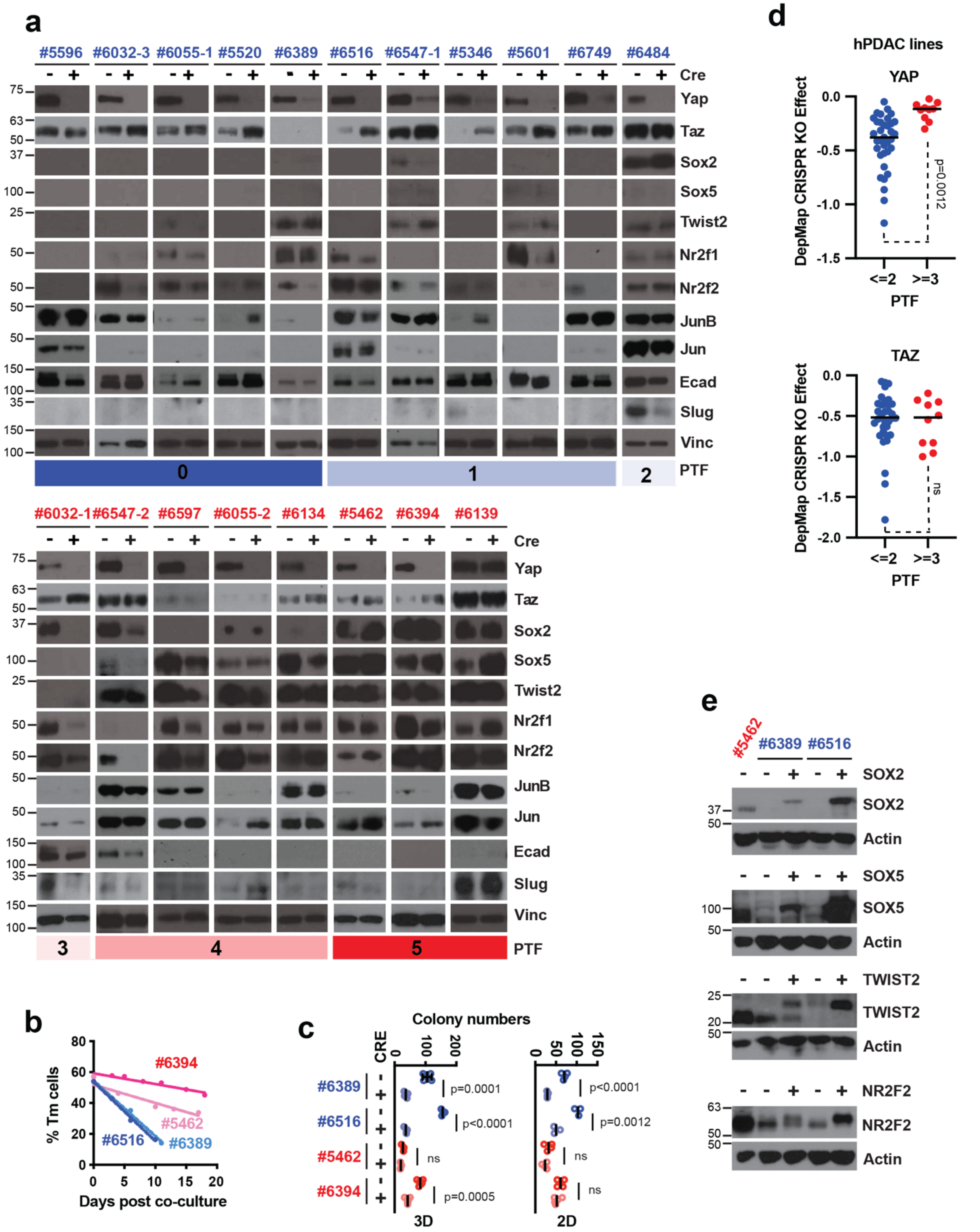

Supplementary Figure 5. PTF expression reduces PDAC cell dependency on Yap. a. WB analysis of primary PDAC lines infected Ad-GFP (-) or Ad-Cre (+) with indicated antibodies. Font colors of mouse IDs represent the differentiation statuses of tumors and corresponding primary PDAC cells (EP: blue; Inter: grey; MS: red). PTF scores (PTF) are indicated by the bottom bars. Vinc was used as loading control. Shown is representative of three independent experiments. **b.** Representative linear regression graphs showing the changes over time in the ratios of Tm+Yap- cells in co-cultures of primary PDAC cells pre-treated with Ad-GFP or Ad-Cre as outlined in **Figure 3a**. **c.** Bar graphs showing average colony numbers from 2D or 3D colony formation assays of the indicated primary PDAC lines infected Ad-GFP (-) or Ad-Cre (+). 3D: #6389 (n=4 independent experiments), #6516 (n=3 independent experiments), #5462 (n=3 independent experiments), and #6394 (n=4 independent experiments) and 2D: #6389 (n=4 independent experiments), #6516 (n=3 independent experiments), #5462 (n=3 independent experiments), #6394 (n=5 independent experiments), respectively. Unpaired, two-tailed, Student's t-test. Data are presented as mean value +/- SEM. **d.** Dot plots showing the KO effects of YAP or TAZ in individual human PDAC cell lines with PTF scores <=2 and >=3 according to the DepMap 22Q2 CRISPR screen. Data are presented as mean value +/- SEM. **e.** WB analysis confirming the exogenous expression of indicated PTFs in #6389 and #6516 cells at comparable levels as their endogenous expression in #5462. Actin was used as loading control. Shown is representative of three independent experiments. Source data are provided as a Source Data file.

Supplementary Figure 6

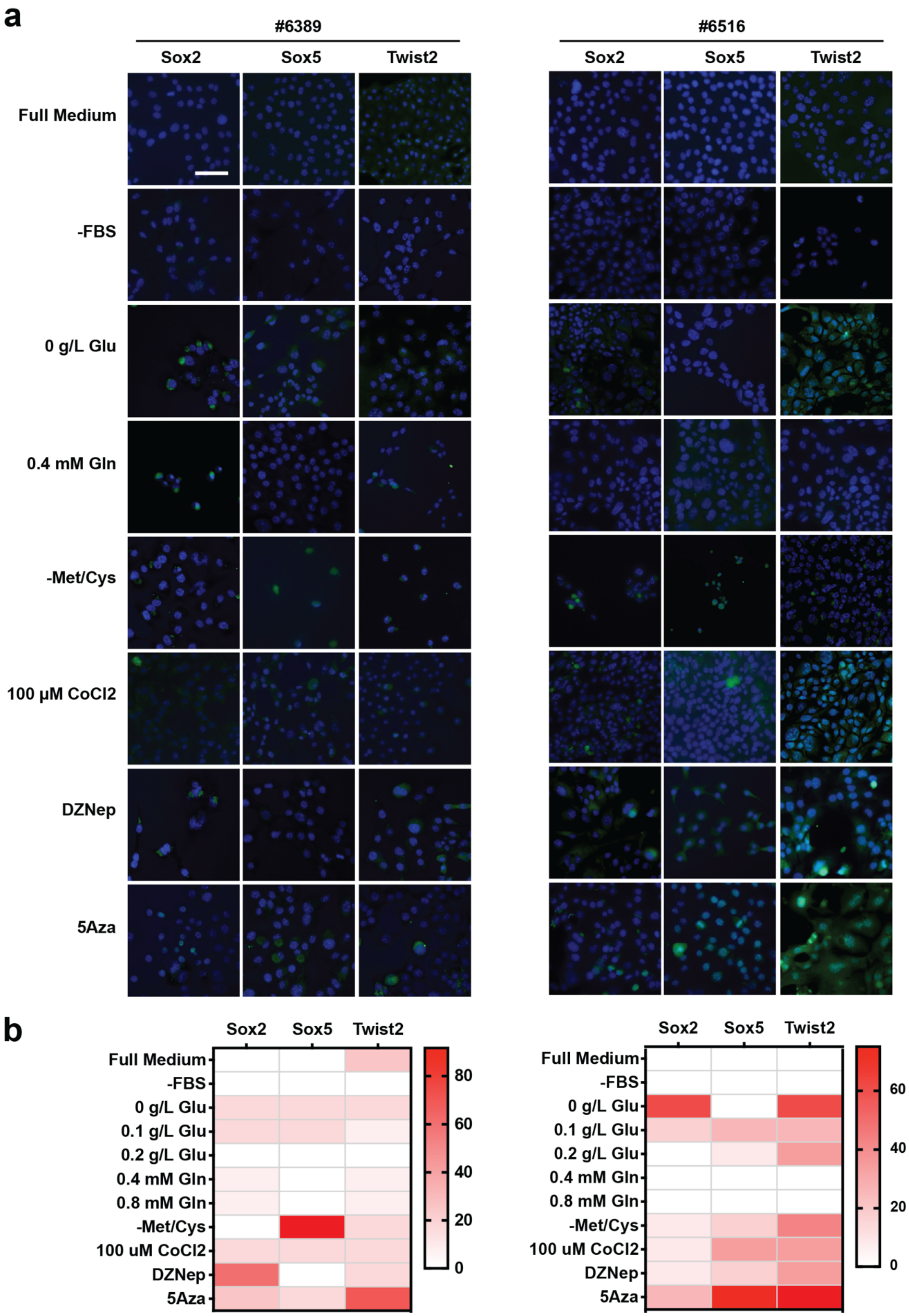

Supplementary Figure 6. Nutrient deprivation, hypoxia and DNA/histone methylation inhibitors induce PTF expression in PDAC cells. Representative images and heatmaps depicting the percentage of randomly selected 10x microscopic fields positive for Sox2, Sox5 or Twist2 according to IF analysis of #6389 and #6516 cells after 2 weeks of culturing under indicated conditions (n = 12 fields per condition). Scale bar indicates 200 µm. Source data are provided as a Source Data file.

Supplementary Figure 7

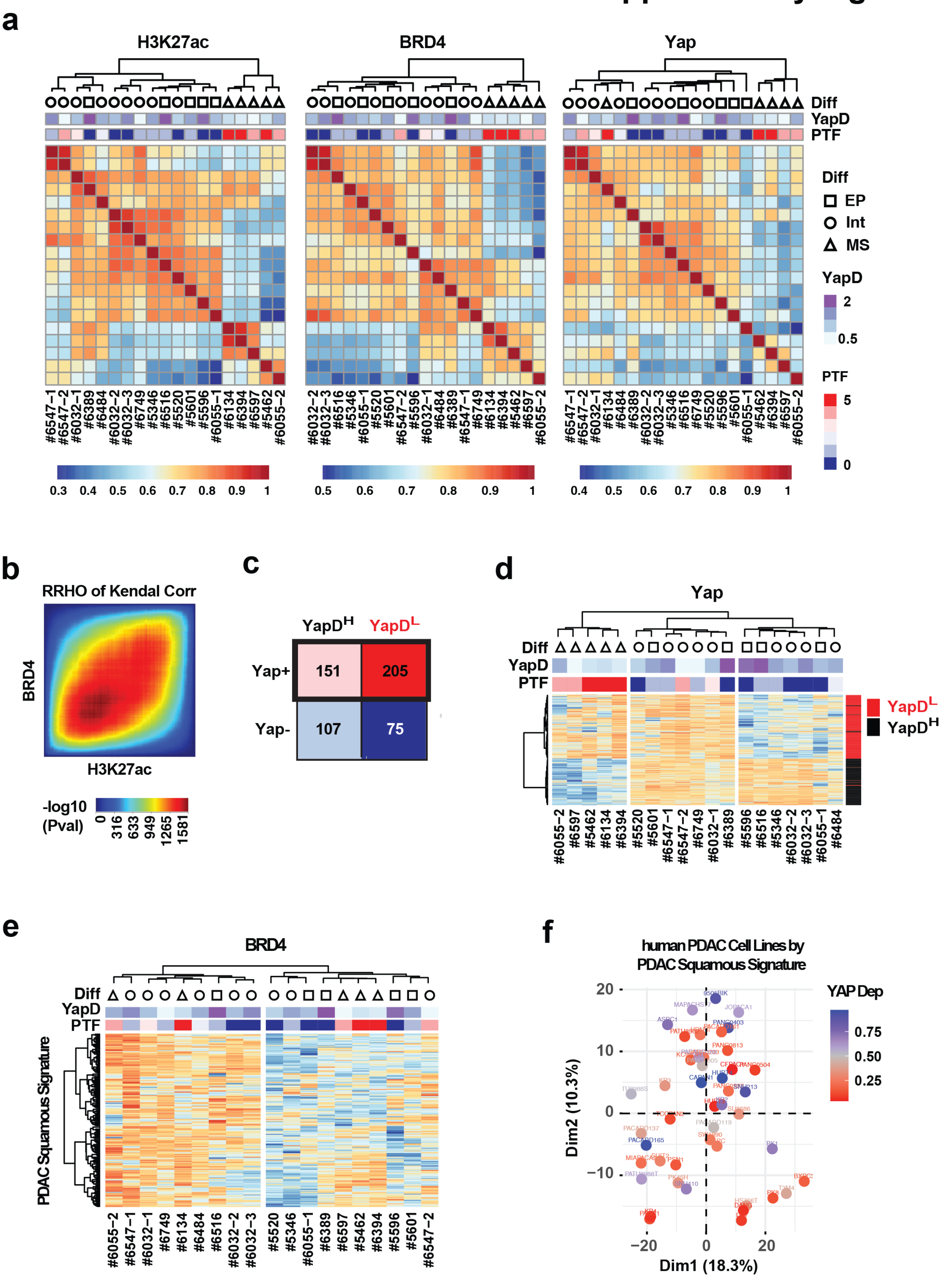

Supplementary Figure /7. Global epigenetic reprogramming associated with EMT is linked to reduced dependency of PDAC cells on Yap. a. Unsupervised clustering of all 19 primary PDAC lines based on pairwise Spearman correlation co-efficiencies of H3K27ac, BRD4 or Yap Cut&Tag signals across the entire genome. **b.** RRHO representation of Kendal correlation co-efficiencies calculated by comparing BRD4 and H3K27ac Cut&Tag signal variations for each genomic locus against the corresponding Yap dependency scores across all 19 primary PDAC lines. **c.** Heatmap depicting the overall Yap-binding statuses at genomic loci associated with either high ($YapD^H$) or low ($YapD^L$) Yap dependency from **Figure 3B**. **d.** Heatmap depicting unsupervised clustering of all 19 primary PDAC lines based on the signals of Yap Cut&Tag peaks overlapping the genomic loci associated with either high ($YapD^H$, black bars, n=151) or low ($YapD^L$, red bars, n=205) Yap dependency from **Figure 4B**. **e.** Heatmap depicting unsupervised clustering of all 19 primary PDAC lines based on the BRD4 Cut&Tag signals at the TSS sites corresponding to the PDAC squamous signature genes defined by Somerville et al. **f.** PCA plots segregating human PDAC lines based on the mRNA expression of PDAC squamous signature genes as shown in **e**. Each PDAC line is represented as a dot colored according to its YAP dependency score from the DepMap 22Q2 CRISPR screen. Source data are provided as a Source Data file.

Supplementary Figure 8

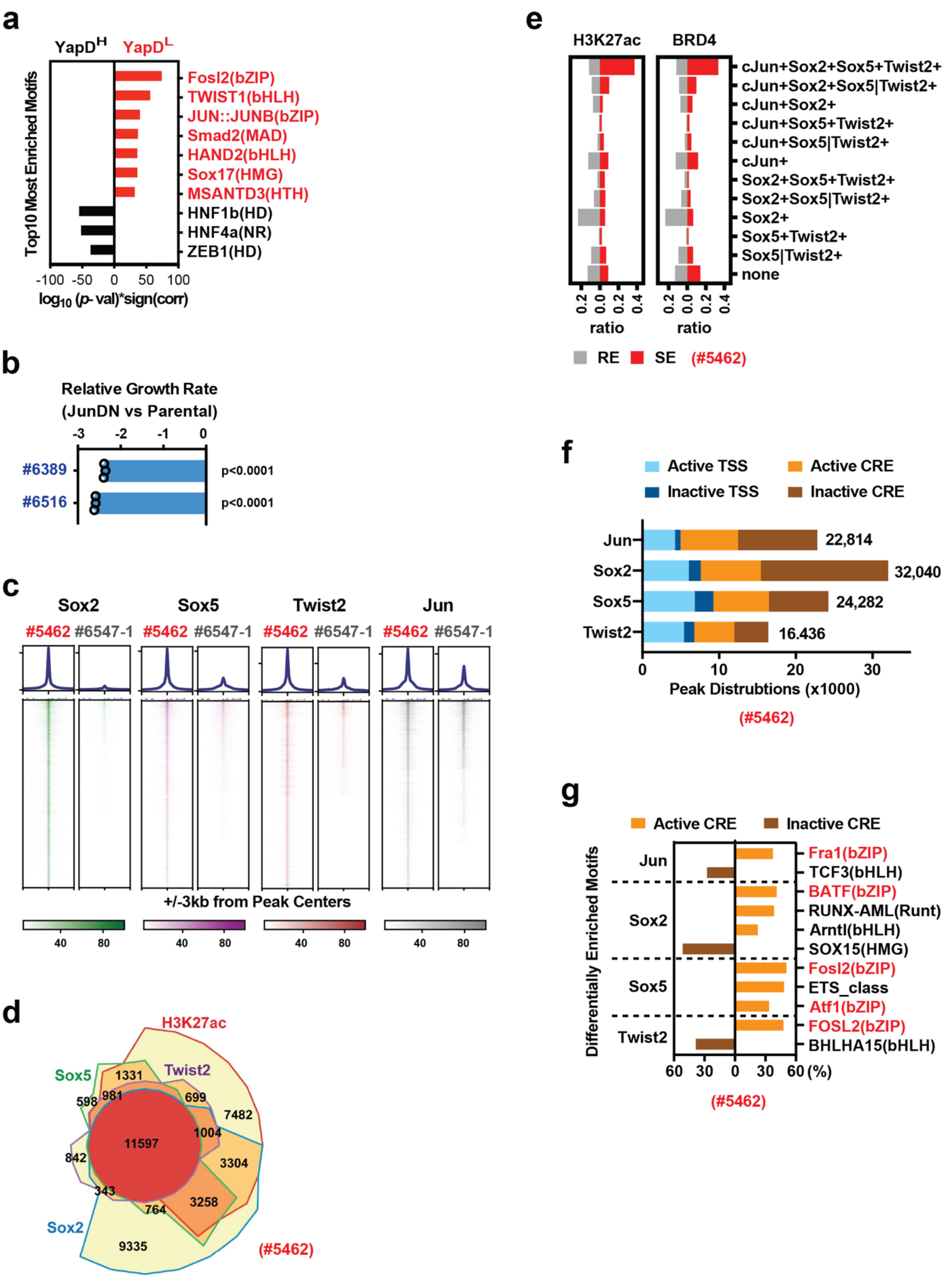

Supplementary Figure 8. PTFs and Jun co-occupy active enhancer sites associated with Yap independency. a. Top10 most differentially enriched motifs according to HOMER differential motif enrichment analysis of BRD4 Cut&Tag peaks overlapping with genomic loci associated with either high ($YapD^{H}$) or low ($YapD^{L}$) Yap dependency from **Figure 4b**. **b.** Linear regression graphs showing the changes over time in the ratios of JunDN cells in co-cultures with parental primary PDAC cells as outlined in **Figure 3a**. n=3 independent experiments. Unpaired, two-tailed, Student's t-test. Data are presented as mean value +/- SEM. **c.** Cut&Tag signal heatmaps from the indicated antibodies within +/-3kb from the peak centers from PTF-high #5462 and PTF-low #6547-1 cells. **d.** Chow-Ruskey Venn diagram of the numbers of overlapping H3K27ac, Sox2, Sox5, and Twist2 Cut&Tag peaks in #5462 cells. **e.** Summary of the ratio of H3K27ac and BRD4 peaks at RE (gray) and SE (red) regions with different binding statuses by Jun, Sox2, Sox5, and/or Twist2 in #5462 cells. **f.** Peak distributions of each TF at active TSS (light blue), inactive TSS (dark blue), active CRE (light brown) and inactive CRE (dark brown) sites. **g.** Percentage of peaks containing the top most differentially enriched motifs according to HOMER differential motif enrichment analysis between Jun, Sox2, Sox5 or Twist2 peaks overlapping with active (light brown) or inactive (dark brown) CRE sites from **f**. Source data are provided within a Source Data file. Cut&Tag data are deposited in the GEO database under accession code GSE224566.

Supplementary Figure 9

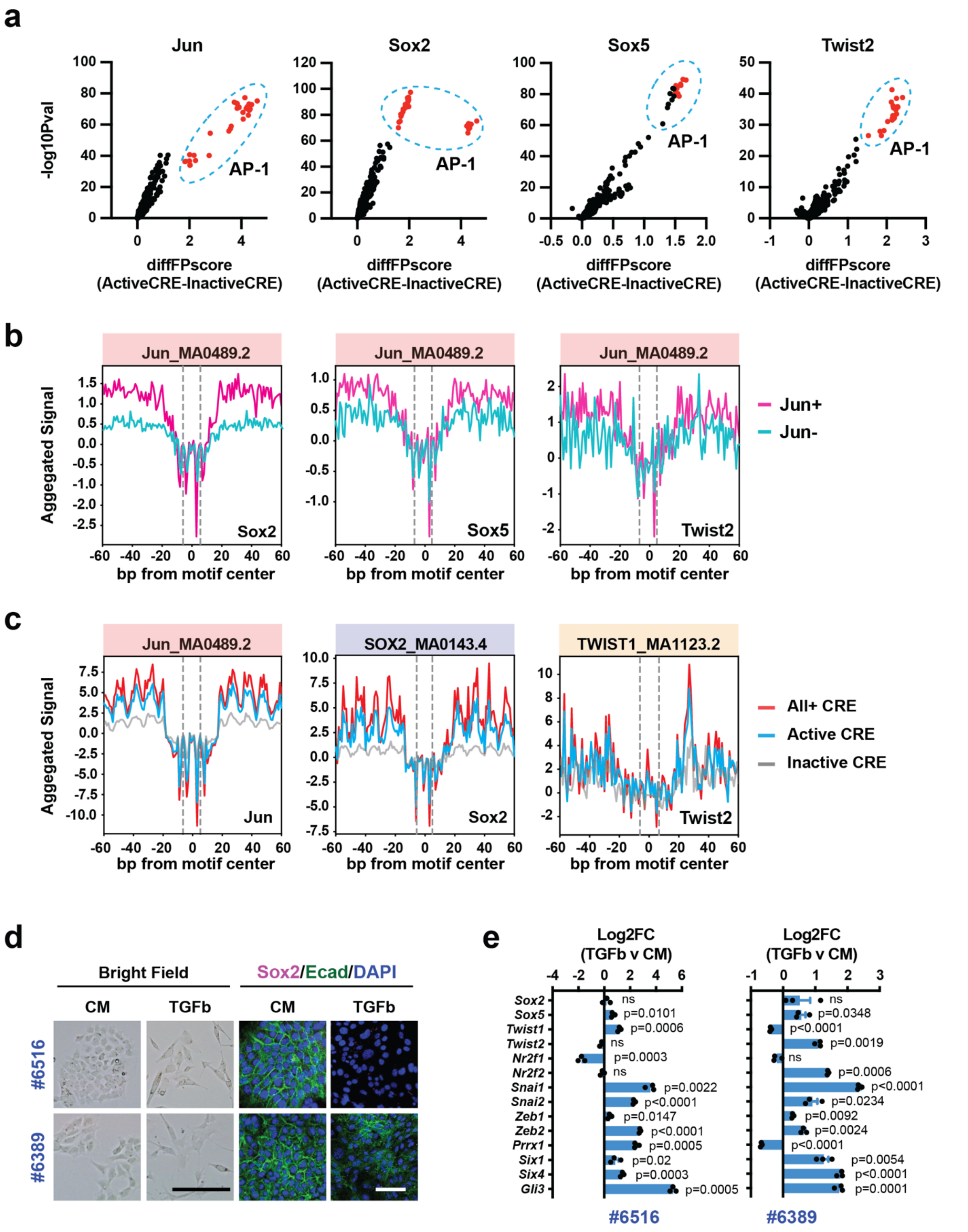

Supplementary Figure 9. PTFs are recruited to AP-1 sites by Jun and cooperate with Jun to activate transcription. **a.** Differential Jun, Sox2, Sox5, and Twist2 footprint scores (diffFPscore) and the corresponding -log10 P-values between active and inactive CRE at the sites of individual JASPAR motifs as determined by TOBIAS. Motifs with diffFPscore greater than 1.5 and -log10 P-values greater than 20 are highlighted in red. Dots corresponding to the AP-1 family of motifs are circled by blue dashed lines. **b.** Aggregated signal centering the Jun_MA0489.2 motif (marked by grey dashed lines) within the Sox2, Sox5 or Twist2 peaks with (+, pink) or without (-, green) Jun co-binding. **c.** Aggregated signal centering the indicated motifs (marked by grey dashed lines) within the Jun, Sox2, or Twist2 peaks at active (blue) or inactive CRE (gray), or CRE bound by Jun and all the PTFs (red). **d.** Representative brightfield (left panel) and IF (right panel) images of Ecad (green) and Sox2 (purple) in indicated primary PDAC cells following extended treatments with TGFb or control medium. Scale bar indicates 50 µm on the left panel and 100 µm on the right panel. Shown is representative images from three independent experiments. **e.** Log2 FC in mRNA expression of indicated genes in indicated PDAC cells treated with TGFb relative to control. n = 3 independent experiments. Unpaired, two-tailed, Student's t-test. Data are presented as mean value +/- SEM. Source data are provided within a Source Data file.

Supplementary Figure 10

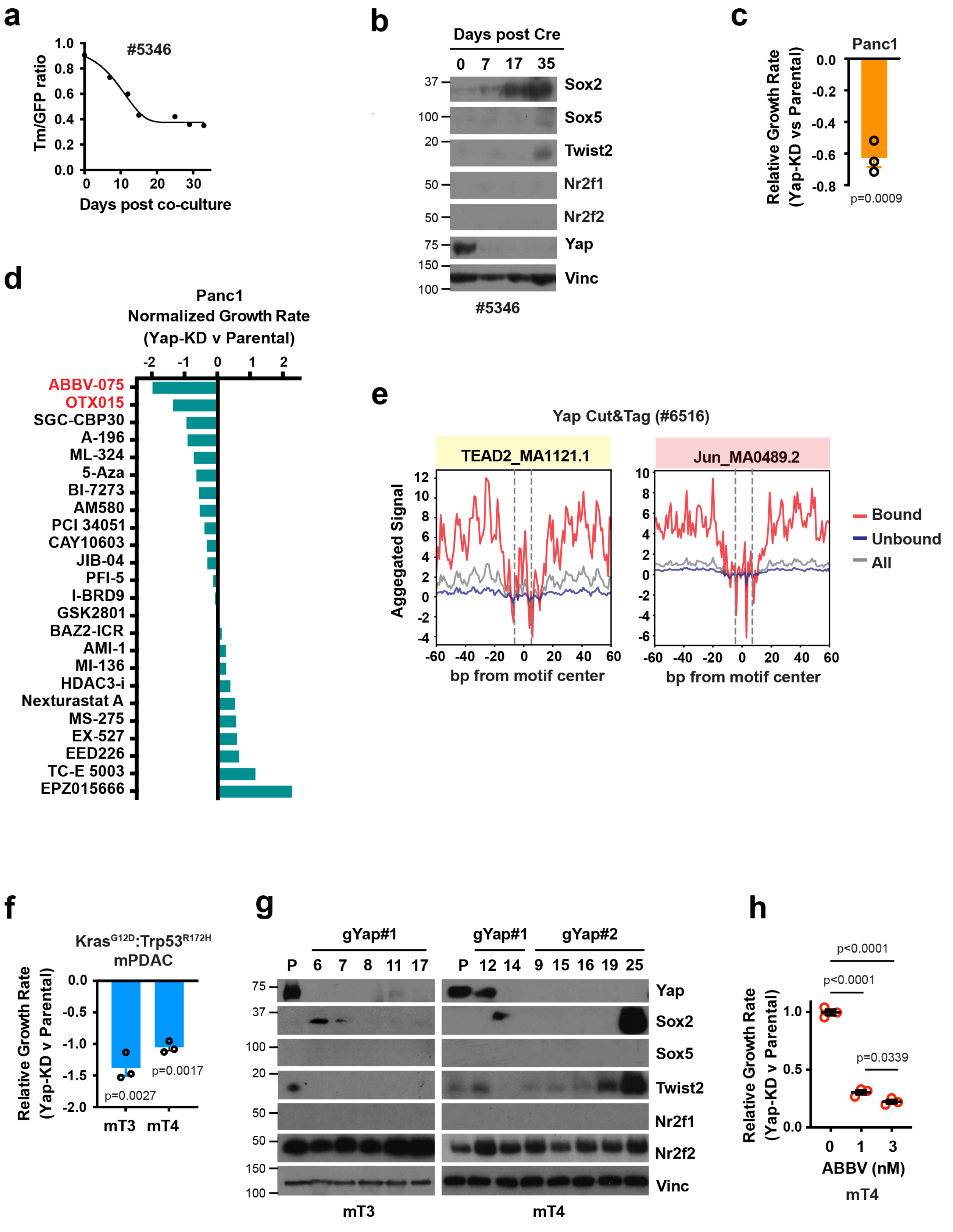

Supplementary Figure 10. BET inhibitors sensitize human and murine PDAC cells to Yap inhibition in vitro and in vivo. a. Changes in the ratios of #5346 cells pretreated with Ad-Cre (Tm+Yap-) or Ad-GFP (GFP+Yap-) and co-cultured over indicated time. **b.** WB of indicated proteins in #5346 cells at the indicated days post infection with Ad-CRE. Vinc was used as the loading control. Shown is representative of three independent experiments. **c.** Log2 relative growth rates of Yap-KD versus parental Panc1 cells. n=3 independent experiments. Unpaired, two-tailed, Student's t-test. Data are presented as mean value +/- SEM. **d.** Log2 relative growth rates of Yap-KD versus parental Panc1 cells co-cultured over a 2-month period in the presence of indicated epigenetic inhibitors normalized to DMSO. BET inhibitors are highlighted with red. **e.** Aggregated signals from bound (red), unbound (blue), or all (gray) Yap peaks centered on the indicated motif sites (marked by grey dashed lines) in #6516 cells. The total numbers of bound, unbound and all peaks for each motif are: TEAD2_MA1121.1:711, 2492 and 3203; Jun_MA0489.2: 798, 5481 and 6279. **f.** Log2 relative growth rates of Yap-KD versus parental mT3 and mT4 cells. n=3 independent experiments. Data are presented as mean value +/- SEM. Unpaired, two-tailed, Student's t-test. **g.** WB analysis of indicated proteins in CRISPR Yap KO clones derived from mT3 and mT4 cells. Shown is representative of three independent experiments. **h.** Relative growth rate of Yap-KD to parental mT4 cells after co-culturing in the presence of indicated concentrations of ABBV-075. n=3 independent experiments. Data are presented as mean value +/- SEM. Unpaired, two-tailed, Student's t-test. Source data are provided within a Source Data file. Cut&Tag data are deposited in the GEO database under accession code GSE224566.

Supplementary Figure 11

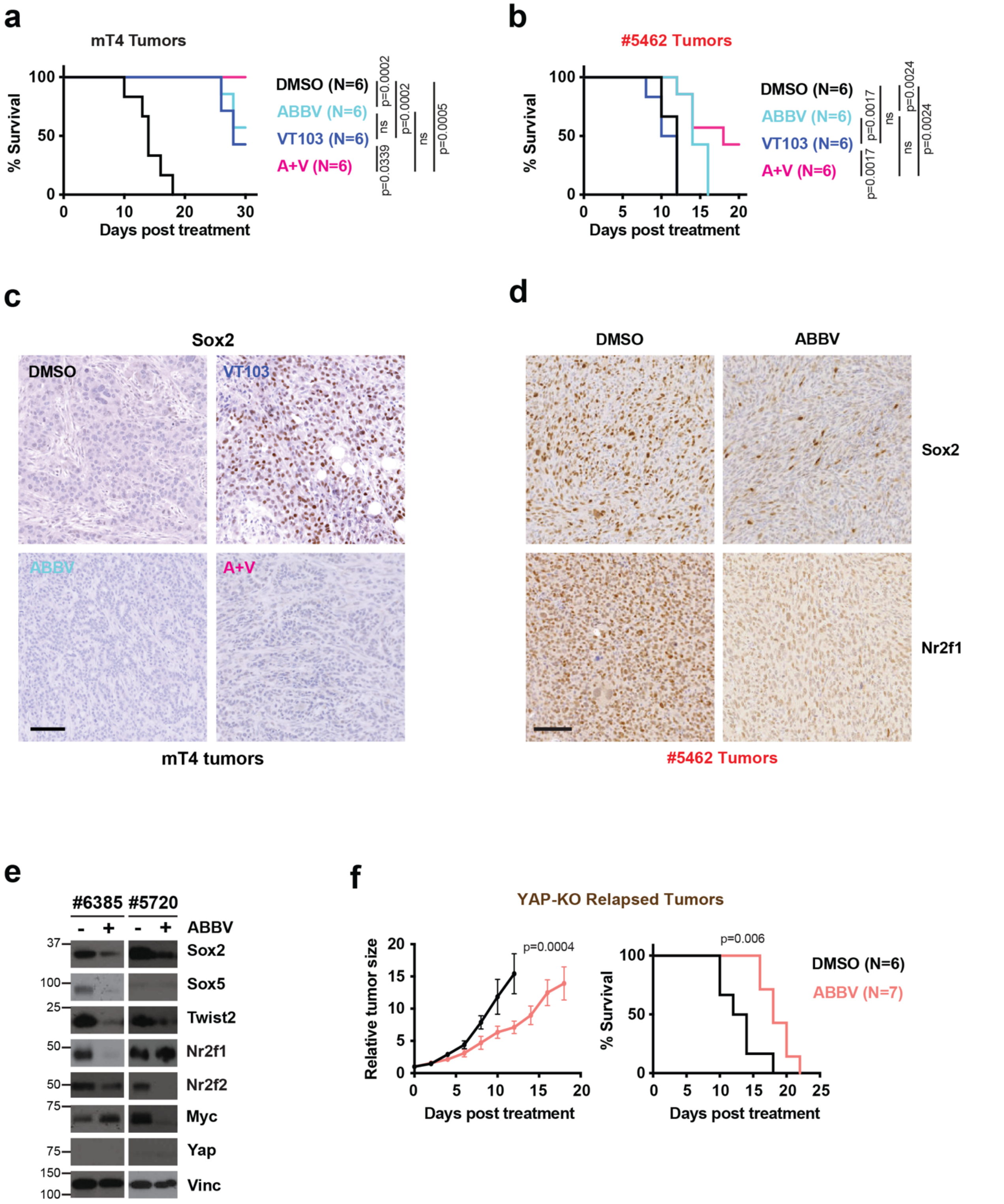

Supplementary Figure 11. BET inhibitor ABBV-075 reduces PTF expression and the growth of Yap-independent murine PDAC tumors. a-b. Kaplan-Meier survival curve of mice carrying subcutaneous xenografts derived from mT4 (**a**) and #5462 cells (**b**) treated with ABBV (light blue), VT103 (blue), ABBV+VT103 (A+V) (pink) or DMSO (black). n = 6 mice for each treatment arm. Log-rank (Mantel-Cox) test. **c-d.** Representative IHC images with indicated antibodies of mT4 (**c**) and #5462 (**d**) xenografts treated with treated with ABBV (light blue), VT103 (blue), ABBV+VT103 (A+V) (pink) or DMSO (black). Experiments were performed on three different mice from each treatment. Scale bar indicates 100 μm. **e.** WB analysis of indicated proteins in two primary PDAC lines derived from relapsed Yap-KO tumors from TAM-treated KPYYF mice. Vinc was used as the loading control. Shown is representative of three independent experiments. **f.** Changes in relative tumor size (left) and overall survival (right) of nude mice bearing #6385 Yap-KO Relapsed xenografts treated with ABBV (red; n=7 mice) or DMSO (black; n=6 mice). Left: Two-way ANOVA test. Right: Log-rank (Mantel-Cox) test. Data are presented as mean value +/- SEM. Source data are provided as a Source Data file.

Supplementary Table 1. The information of mice

Tumor_ID	Mouse_ID	Gender	Genotype	Age (days)	TAM (days)
#6028-T00	#6028	Female	KPF	121	4
#6028-T01	#6028	Female	KPF	121	4
#6114-T00	#6114	Female	KPF	41	6
#4921-T00	#4921	Female	KPF	61	9
#4921-T01	#4921	Female	KPF	61	9
#4921-T02	#4921	Female	KPF	61	9
#4921-T03	#4921	Female	KPF	61	9
#4921-T04	#4921	Female	KPF	61	9
#6149-T00	#6149	Female	KPF	116	11
#5997-T00	#5997	Female	KPF	143	23
#5897-T00	#5897	Female	KPF	149	30
#5996-T00	#5996	Male	KPF	152	32
#4785-T00	#4785	Male	KPF	106	36
#4785-T01	#4785	Male	KPF	106	36
#4785-T02	#4785	Male	KPF	106	36
#4785-T03	#4785	Male	KPF	106	36
#4943-T00	#4943	Male	KPF	149	79
#5376-T00	#5376	Male	KPYF	149	40
#4808-T00	#4808	Female	KPYF	77	43
#5379-T00	#5379	Male	KPYF	178	69
#4852-T00	#4852	Male	KPYF	150	72
#5024-T00	#5024	Female	KPYF	174	94
#5136-T00	#5136	Male	KPYF	178	102
#4892-T00	#4892	Female	KPYYF ($Tm^{H}Yap^{KO}$)	122	36
#8224-T00	#8224	Male	KPYYF ($Tm^{H}Yap^{KO}$)	109	48

#4449-T00	#4449	Female	KPYYF (Tm^HYap^{KO})	126	51
#7783-T00	#7783	Female	KPYYF (Tm^HYap^{KO})	129	79
#7783-T01	#7783	Female	KPYYF (Tm^HYap^{KO})	129	79
#6385-T00	#6385	Male	KPYYF (Tm^HYap^{KO})	222	132
#6385-T01	#6385	Male	KPYYF (Tm^HYap^{KO})	222	132
#7641-T00	#7641	Female	KPYYF (Tm^HYap^{KO})	197	145
#7641-T01	#7641	Female	KPYYF (Tm^HYap^{KO})	197	145
#4697-T00	#4697	Female	KPYYF (Tm^HYap^{KO})	246	178
#4697-T01	#4697	Female	KPYYF (Tm^HYap^{KO})	246	178
#4697-T02	#4697	Female	KPYYF (Tm^HYap^{KO})	246	178
#4697-T03	#4697	Female	KPYYF (Tm^HYap^{KO})	246	178
#5720-T00	#5720	Female	KPYYF ($Tm\text{-}Yap^{KO}$)	161	27
#4455-T00	#4455	Female	KPYYF (Tm^HYap^L)	61	6
#6387-T00	#6387	Female	KPYYF (Tm^HYap^L)	43	7
#5601-T00	#5601	Male	KPYYF (Tm^HYap^L)	159	17
#5601-T01	#5601	Male	KPYYF (Tm^HYap^L)	159	17
#4641-T00	#4641	Female	KPYYF (Tm^HYap^L)	63	26
#4641-T01	#4641	Female	KPYYF (Tm^HYap^L)	63	26
#4891-T00	#4891	Female	KPYYF (Tm^HYap^L)	115	29
#4938-T00	#4938	Female	KPYYF (Tm^HYap^L)	128	29
#6365-T00	#6365	Male	KPYYF (Tm^HYap^L)	140	40
#6365-T01	#6365	Male	KPYYF (Tm^HYap^L)	140	40
#5522-T01	#5522	Male	KPYYF (Tm^HYap^L)	136	41
#6928-T00	#6928	Female	KPYYF (Tm^HYap^L)	127	58
#6928-T01	#6928	Female	KPYYF (Tm^HYap^L)	127	58
#4516-T01	#4516	Male	KPYYF (Tm^HYap^L)	150	80
#4610-T01	#4610	Female	KPYYF (Tm^HYap^L)	205	141
#6821-T00	#6821	Male	KPYYF (Tm^LYap^H)	80	2

#5346-T00	#5346	Male	KPYYF ($Tm^L Yap^H$)	127	10
#5596-T00	#5596	Female	KPYYF ($Tm^L Yap^H$)	155	13
#5522-T00	#5522	Male	KPYYF ($Tm^L Yap^H$)	136	41
#7369-T00	#7369	Male	KPYYF ($Tm^L Yap^H$)	133	67
#4516-T00	#4516	Male	KPYYF ($Tm^L Yap^H$)	150	80
#4610-T00	#4610	Female	KPYYF ($Tm^L Yap^H$)	205	141

Supplementary Table 2. The HOMER motifs whose genomic locations were used to plot Sox2, Sox5, Twist2 and Jun Cut&Tag signals in Figure 5b.

Homer Motif Name	Homer Motif Profile
Sox2(HMG)/mES-Sox2-ChIP-Seq(GSE11431)/Homer (Motif 338)	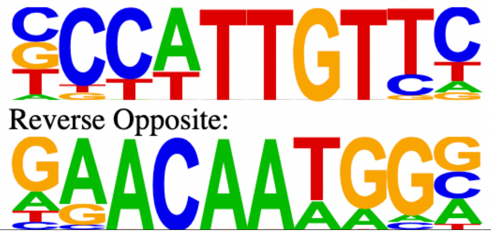
Sox6(HMG)/Myotubes-Sox6-ChIP-Seq(GSE32627)/Homer (Motif 341)	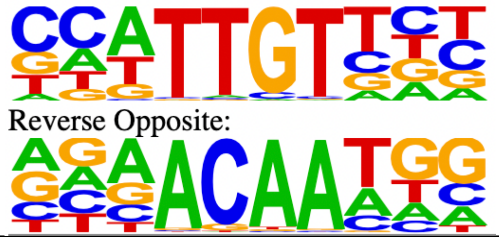
Twist2(bHLH)/Myoblast-Twist2.Ty1-ChIP-Seq(GSE127998)/Homer (Motif 391)	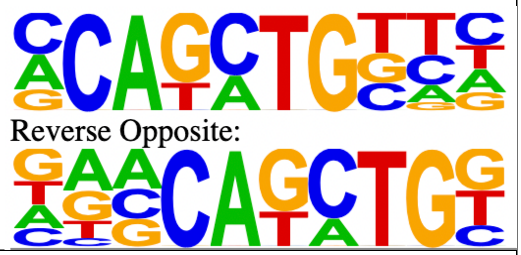
AP-1(bZIP)/ThioMac-PU.1-ChIP-Seq(GSE21512)/Homer (Motif 1)	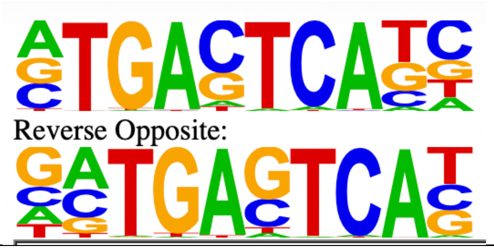

Supplementary Table 3. The list of epigenetic inhibitors

Inhibitor	Concentration (μM)	
	mPDAC cells	Panc1
3-Deazaneplanoci (DZNep)	1	None
5-Azacytidine	2	0.66
A-196	10	2.5
ABBV-075	0.005	0.11
AM580	20	20
AMI-1	0.5	10
AR-42 (OSU-HDAC42)	1	None
BAZ2-ICR	25	25
BI-7273	1	20
BMS 493	5	None
BRD4884	1.88	2.5
CAY10603	1	0.022
EED226	20	6.66
EPZ015666	20	2.5
EPZ6438	1	None
EX-527	30	2.5
GSK126	2	None
GSK2801	15	15
GSK6853	20	None
HDAC3 inhibitor	1	20
I-BRD9	15	10
JIB-04	0.375	0.037
LE 135	4	None
MI-136	3.33	2.5
ML-324	0.83	1.66
MM-102	0.5	None
MS-275	10	1
MS023	5	None
Nexturastat A	2.5	1
OTX015	0.25	0.1
Panobinostat	0.5	None
PCI 24781	0.4	0.125
PCI 34051	20	20
PFI-3	30	None
PFI-5	15	15
PRT4165	72	None

PTC-209	0.5	None
SGC-CBP30	18	3
SGC707	12.5	None
TC-E 5002	30	30
TC-E 5003	0.75	0.75
Trichostatin A (TSA)	0.2	None
UNC0379	6.25	2.5

Supplementary Table 4. The list of reagents and resources

REAGENT or RESOURCE	SOURCE	IDENTIFIER
**Antibodies**		
Chicken polyclonal anti-GFP	Abcam Cat#ab13970; RRID:AB_300798	IHC 1:2000
Goat polyclonal anti-tdTomato	LifeSpan Cat#LS-C348313	IHC 1:2000
Ki67 Rabbit polyclonal antibody	MilliporeSigma Cat#ab9260; AB_RRID:2142366	IHC 1:500
HNF-4 alpha/NR2A1 Rabbit polyclonal antibody	NOVUS BIOLOGICALS Cat#NBP1-89679	IHC 1:1000
CDH2 rat mAb	DSHB Cat#MNCD2; RRID:AB_528119	IHC 1:200
Mouse monoclonal Anti-E-Cadherin (clone36)	BD Biosciences Cat#610182; RRID:AB_397581	IHC 1:2000; IF 1:500; WB 1:2000
Mouse monoclonal anti-Vinculin (Clone 7F9)	Santa Cruz Biotechnology Cat#sc-73614; RRID:AB_1131294	WB 1:2000
Mouse monoclonal anti-β-Actin (Clone C4)	Santa Cruz Biotechnology Cat#sc-47778; RRID:AB_2714189	WB 1:5000
ZEB1 (E2G6Y) XP Rabbit mAb	Cell Signaling Technology Cat#70512	IHC 1:500

JunB (C37F9) Rabbit mAb	Cell Signaling Technology Cat#3753; RRID:AB_2130002	IHC 1:500; WB 1:1000
Rabbit monoclonal anti-c-Myc (D3N8F)	Cell Signaling Technology Cat#13987; RRID:AB_2631168	WB 1:1000
Rabbit polyclonal anti-YAP	Cell Signaling Technology Cat#4912; RRID:AB_2218911	IHC 1:500; WB 1:1000; Cut&Tag 1:50
Rabbit monoclonal anti-SOX2 (Clone D6D9)	Cell Signaling Technology Cat#5024; RRID:AB_1904142	IHC 1:500; IF 1:200; WB 1:1000; Cut&Tag 1:50
SOX5 Polyclonal antibody	Proteintech Cat#13216-1-AP; RRID:AB=2196089	IHC 1:500; IF 1:200; WB 1:1000; Cut&Tag 1:50
TWIST2 Polyclonal antibody	Proteintech Cat#11752-1-AP; RRID:AB_2877791	IHC 1:500; IF 1:200; WB 1:1000; Cut&Tag 1:100
COUP-TFI (D4H2) Rabbit mAb antibody (NR2F1)	Cell Signaling Technology Cat#6364; RRID:AB_11220432	IHC 1:500; WB 1:1000
COUP-TFII (D16C4) Rabbit mAb (NR2F2)	Cell Signaling Technology Cat#6434; RRID:AB_11220428	IHC 1:500; WB 1:1000
ETS-1 (D8O8A) Rabbit mAb	Cell Signaling Technology Cat#14069	WB 1:1000
MAF polyclonal antibody	Proteintech Cat#55013-1-AP; RRID:AB_10863127	WB 1:1000

SIX4 polyclonal antibody	Proteintech Cat#21305-1-AP; RRID:AB_10860258	WB 1:1000
PRX (Prrx1; C-6) antibody	Santa Cruz Biotechnology Cat#sc-271047; RRID:AB_10611937	WB 1:1000
p40 Mouse monoclonal antibody	BIOCARE MEDICAL Cat#ACI 3066 A; RRID:AB_2858274	IHC 1:500
Rat monoclonal anti-Keratin, type I; cytokeratin 19	DSHB Cat#TROMA-III; RRID:AB_2133570	IHC 1:500; WB 1:2000
Rabbit Anti-BRD4 antibody	Sigma-Aldrich Cat#HPA015055; RRID:AB_1845435"	Cut&Tag 1:50
Anti-acetyl-Histone H3 (Lys27) Antibody	MilliporeSigma Cat#07-360; RRID:AB_310550	Cut&Tag 1:50
Anti-trimethyl-Histone H3 (Lys4) Antibody	MilliporeSigma Cat#07-473; RRID:AB_1977252	Cut&Tag 1:100
c-Jun (60A8) Rabbit mAb antibody	Cell Signaling Technology Cat#9165; RRID:AB_2130165	WB 1:1000; Cut&Tag 1:100
Anti-rabbit IgG, HRP-linked Antibody	Cell Signaling Technology Cat#7074	WB 1:5000
Anti-mouse IgG, HRP-linked Antibody	Cell Signaling Technology Cat#7076	WB 1:5000
**Chemicals, Peptides, and Recombinant Proteins**		
5-Azacytidine	ApexBio	Cat#A1907; CAS: 320-67-2
ABBV-075	Cayman	Cat#21033; CAS: 1445993-26-9

OTX015	Cayman	Cat#15947; CAS: 202590-98-5
JQ1	Cayman	Cat#11187; CAS: 1268524-70-4
ML-324	Cayman	Cat#17472; CAS: 1222800-79-4
PRT4165	Cayman	Cat#19093; CAS: 31083-55-3
MI-136	Cayman	Cat#19245; CAS: 1628316-74-4
TC-E 5003	Cayman	Cat#17718; CAS: 17328-16-4
PFI-3	Cayman	Cat#15267; CAS: 1819363-80-8
UNC0379	Cayman	Cat#16400; CAS: 1620401-82-2
PFI-5	Cayman	Cat#15267; CAS: 1819363-80-8
Trichostatin A	Cayman	Cat#89730; CAS: 58880-19-6
GSK126	Cayman	Cat#15415; CAS: 1346574-57-9
GSK484	Cayman	Cat#17488; CAS: 1652591-81-5
MM-102	Cayman	Cat#17699; CAS: 1417329-24-8
TP-472	Cayman	Cat#20030; CAS: 2079895-62-6
BRD4884	Cayman	Cat#19834; CAS: 1404559-91-6
BMS 453	Cayman	Cat#19076; CAS: 166977-43-1
UNC3866	Cayman	Cat#19237; CAS: 1872382-47-2
CD2665	Cayman	Cat#16031; CAS: 170355-78-9

MI192	Cayman	Cat#18288; CAS: 1415340-63-4
DZNep	Cayman	Cat#13828; CAS: 102052-95-9
TC-E 5002	Cayman	Cat#17717; CAS: 1453071-47-0
(R)-PFI-2	Cayman	Cat#14678; CAS: 1627607-87-7
EPZ5676	Cayman	Cat#16175; CAS: 1380288-87-8
BAY-6035	Cayman	Cat#25925; CAS: 2247890-13-5
UNC1215	Cayman	Cat#13968; CAS: 1415800-43-9
C646	Cayman	Cat#10549; CAS: 328968-36-1
JIB-04	Cayman	Cat#15338; CAS: 199596-05-9
CAY10603	Cayman	Cat#13146; CAS: 1045792-66-2
Furamidine	Cayman	Cat#19121; CAS: 55368-40-6
BI-7273	Cayman	Cat#20311; CAS: 1883429-21-7
SGC3027	SGC	Cat#6825; CAS:None
GSK-LSD1	Cayman	Cat#16439; CAS: 2102933-95-7
MRK-740	MedChemExpress	Cat#HY-114209; CAS: 2387510-80-5
NI-57	Cayman	Cat#17662; CAS: 1883548-89-7
GSK6853	Cayman	Cat#20985; CAS: 1910124-24-1
AR-42	Cayman	Cat#17531; CAS: 1798310-55-0

AM580	Cayman	Cat#15261; CAS: 102121-60-8
BIX 01294	Cayman	Cat#13124; CAS: 1808255-64-2
OICR-9429	Cayman	Cat#16095; CAS: 1801787-56-3
BAZ2-ICR	Cayman	Cat#17448; CAS: 1665195-94-7
EED226	Cayman	Cat#22031; CAS: 2083627-02-3
EPZ015666	Cayman	Cat#17285; CAS: 1616391-65-1
EPZ020411	Cayman	Cat#19160; CAS: 1700663-41-7
PTC-209	Cayman	Cat#16277; CAS: 315704-66-6
MS-275	Cayman	Cat#13284; CAS: 209783-80-2
TMP269	Cayman	Cat#17738; CAS: 1314890-29-3
EX-527	Cayman	Cat#10009798; CAS: 49843-98-3
SGC707	Cayman	Cat#17017; CAS: 1687736-54-4
AMI-1	Cayman	Cat#13965; CAS: None
BMS493	Cayman	Cat#17418; CAS: 215030-90-3
Panobistat	MedChemExpress	Cat#HY-10224; CAS: 404950-80-7
PCI 34051	Cayman	Cat#10444; CAS: 950762-95-5
A-196	Cayman	Cat#18317; CAS: 1982372-88-2
LE 135	Cayman	Cat#14415; CAS: 155877-83-1

Nextrastat A	Cayman	Cat#71462653; CAS No.1403783-31-2
GSK2801	Cayman	Cat#14120; CAS: 1619994-68-1
GSK-J4	Cayman	Cat#12073; CAS: 1797983-09-5
MS023	Cayman	Cat#34786; CAS: 1831110-54-3
BRD73954	Cayman	Cat#16919; CAS: 1440209-96-0
PCI24781	Cayman	Cat#20059; CAS: 783355-60-2
SGC-CBP30	Cayman	Cat#14469; CAS: 1613695-14-9
I-BRD9	Cayman	Cat#17749; CAS: 1714146-59-4
EPZ6438	Cayman	Cat#16174; CAS: 1403254-99-8
Vivace102 (V102)	Vivace Therapeutics	None
Puromycin	Thermo Fisher Scientific	Cat# A1113803
Doxycycline hydrochloride	Alfa Aesar	Cat#J60422; CAS: 10592-13-9
TGFb1	MedChemExpress	HY-P7117
**Critical Commercial Assays**		
GoTaq® Green Master Mix	Promega	Cat#M7122
ImmPACT DAB EqV HRP substrate	Vector Laboratories	Cat#SK-4103
IMMPRESS HRP Polymer Detection Kit	Vector Laboratories	Cat#MP-7401, MP-7402, MP-7405
iScript cDNA Synthesis Kit	Bio-Rad	Cat#1708890
iTaq Universal SYBR Green Supermix	Bio-Rad	Cat#1725124

Pierce BCA Protein Assay Kit	Thermo Fisher Scientific	Cat#23225
PrepEase Genomic DNA isolation kit	Affymetrix	Cat#78855
RNAeasy Mini Kit	Qiagen	Cat#74104
Tyramide Signal Amplification (TSA) kit	Thermo Fisher Scientific	Cat#T20932, T20933, T20936
NEBNext HiFi 2x PCR Master mix	NEB	Cat#M0541
BioMag®Plus Concanavalin A	Bangs Laboratories	Cat#BP531
Mag-Bind® TotalPure NGS beads	Omega Bio Tech	Cat#M1378
**Experimental Models: Cell Lines**		
Panc1	ATCC	CRL-1469
293T	ATCC	CRL-3216
mT3 and mT4	Sylvia F.Boj ea al., 2015	N/A
Primary mPDAC (KP)	This paper	N/A
Primary mPDAC (KPYY)	This paper	N/A
**Experimental Models: Organizms/Strains**		
Athymic Nude Mouse	Charles River Laboratories (Croy and Chapeau, 1990)	Strain code #553
CL57/BL6 mice	Charles River Laboratories (Croy and Chapeau, 1990)	Strain code #027
Genetically engineered mouse strains	(Schönhuber et al., 2014 (Zhang et al., 2010)	N/A
**Recombinant DNA**		
pTRIPZ lentiviral vector	GE Healthcare Dharmacon, Inc.	Cat#RHS4740
LentiCrispr v2	Addgene	Cat#52961
pCW57-MCS1-2A-MCS2	Addgene	Cat##71782

Ad-CMV-GFP	Iowa viral vector core	Cat#Iowa-1174
Ad-CMV-CRE	Iowa viral vector core	Cat#Iowa-5
pLenti6.2-V5-TWIST2	DNASU	Cat#HsCD00330331
pLX304-SOX2	DNASU	Cat#HsCD00436328
pLX304-SOX5	DNASU	Cat#HsCD00442638
pDNR-Dual-NR2F2	DNASU	Cat#HsCD00005215
pCW57-SOX2	This paper	N/A
pCW57-SOX5	This paper	N/A
pCW57-TWIST2	This paper	N/A
pCW57-NR2F2	This paper	N/A
pTRIPZ-shSox2	This paper	N/A
pTRIPZ-shSox5#1	This paper	N/A
pTRIPZ-Sox5#2	This paper	N/A
pTRIPZ-shTwist2#1	This paper	N/A
pTRIPZ-shTwist2#2	This paper	N/A
pTRIPZ-shNr2f1#1	This paper	N/A
pTRIPZ-shNr2f1#2	This paper	N/A
pTRIPZ-shNr2f2#1	This paper	N/A
pTRIPZ-shNr2f2#2	This paper	N/A
TLCV2-sgNr2f2#1	This paper	N/A
TLCV2-sgNr2f2#2	This paper	N/A
3XFlag-pA-Tn5	Addgene	Cat#124601
**Oligonucleotides**		
Mouse Sox2_shRNA 5' CGAGATAAACATGGCAATCAA 3'	Broad TRC RNAi shRNA library	TRCN0000085748
Mouse Sox5_shRNA 5' ATGGAAGTCGATGGCAATAAA 3'	Broad TRC RNAi shRNA library	TRCN0000421409

Mouse Twist2_shRNA 5' AGCAAGAAATCGAGCGAAGAT 3'	Broad TRC RNAi shRNA library	TRCN0000086085
Mouse Nr2f1_shRNA 5' GTCCGCAGGAACTTAACTTAC 3'	Broad TRC RNAi shRNA library	TRCN0000350649
Mouse Nr2f2_shRNA 5' CTCGTACCTGTCCGGATATAT 3'	Broad TRC RNAi shRNA library	TRCN0000054475
