# Supplementary material for: Spontaneously evolved progenitor niches escape Yap oncogene addiction in advanced pancreatic ductal adenocarcinomas
Source: Nat Commun. 2023 Mar 15;14:1443. doi: 10.1038/s41467-023-37147-y (PMC10017707; doi:10.1038/s41467-023-37147-y)
Supplement: Supplementary file 2 — Description of Additional Supplementary Files [file 41467_2023_37147_MOESM2_ESM.pdf]

Description of Additional File name: Supplementary Files

File name: **Supplementary Software 1**

Description: Row code used in RNA-seq and Cut&Tag data analysis.
