# Supplementary material for: Spontaneously evolved progenitor niches escape Yap oncogene addiction in advanced pancreatic ductal adenocarcinomas
Source: Nat Commun. 2023 Mar 15;14:1443. doi: 10.1038/s41467-023-37147-y (PMC10017707; doi:10.1038/s41467-023-37147-y)
Supplement: Supplementary file 4 — Reporting Summary [file 41467_2023_37147_MOESM4_ESM.pdf]

nature portfolio

Corresponding author(s): Chunling Yi

Last updated by author(s): Mar 2, 2023

Reporting Summary

Nature Portfolio wishes to improve the reproducibility of the work that we publish. This form provides structure for consistency and transparency in reporting. For further information on Nature Portfolio policies, see our Editorial Policies and the Editorial Policy Checklist.

Statistics

For all statistical analyses, confirm that the following items are present in the figure legend, table legend, main text, or Methods section.

n/a	Confirmed	
☐	☒	The exact sample size (*n*) for each experimental group/condition, given as a discrete number and unit of measurement
☐	☒	A statement on whether measurements were taken from distinct samples or whether the same sample was measured repeatedly
☐	☒	The statistical test(s) used AND whether they are one- or two-sided<br>*Only common tests should be described solely by name; describe more complex techniques in the Methods section.*
☒	☐	A description of all covariates tested
☐	☒	A description of any assumptions or corrections, such as tests of normality and adjustment for multiple comparisons
☐	☒	A full description of the statistical parameters including central tendency (e.g. means) or other basic estimates (e.g. regression coefficient) AND variation (e.g. standard deviation) or associated estimates of uncertainty (e.g. confidence intervals)
☐	☒	For null hypothesis testing, the test statistic (e.g. *F*, *t*, *r*) with confidence intervals, effect sizes, degrees of freedom and *P* value noted<br>*Give P values as exact values whenever suitable.*
☐	☒	For Bayesian analysis, information on the choice of priors and Markov chain Monte Carlo settings
☐	☒	For hierarchical and complex designs, identification of the appropriate level for tests and full reporting of outcomes
☐	☒	Estimates of effect sizes (e.g. Cohen's *d*, Pearson's *r*), indicating how they were calculated

Our web collection on statistics for biologists contains articles on many of the points above.

Software and code

Policy information about availability of computer code

Data collection	IHC images of PDAC samples were scanned using The Hamamatsu NanoZoomer slide scanning system.<br>Fluorescence images of PDAC samples were acquired using Leica SP8 confocal microscope.<br>Cells treated with epigenetic inhibitors were stained and measured using Synergy™ H4 Hybrid Multi-Mode Microplate Reader.<br>The percentage of GFP and Tm positive cells were measured by FACS LSRFortessa Cell analyzer.
Data analysis	A Graphpad Prism 9 (v9.5.0) for statical analysis.<br>NDP.view2 Viewing software (v2.9) and image J (v2.9.0) for processing and analyzing IHC images.<br>Synergy score were calculated by SynergyFinder 1.0.<br>FCS express 7 were used to analyze the data from flow cytometry.<br>Hi-Seq data were analyzed by using various packages including GREAT version 3.0.0, GeneSetClustering, R 4.2.0, TrimGalore (v 0.6.5), Bowtie2 (v2.4.5), Picard tools, HOMER (v4.11), csaw (v3.15), deepTools (v2.0), DESeq2 (v3.15), GSEA Java-based software package (v1.60.0), RRHO" r packages (v1.38.0), and TOBIAS (v0.15.0).<br>All the source code and software used for the analysis performed for this study is available in Supplementary Software 1.

For manuscripts utilizing custom algorithms or software that are central to the research but not yet described in published literature, software must be made available to editors and reviewers. We strongly encourage code deposition in a community repository (e.g. GitHub). See the Nature Portfolio guidelines for submitting code & software for further information.

Data

Policy information about availability of data

All manuscripts must include a data availability statement. This statement should provide the following information, where applicable:

- Accession codes, unique identifiers, or web links for publicly available datasets
- A description of any restrictions on data availability
- For clinical datasets or third party data, please ensure that the statement adheres to our policy

The raw fastq data generated from RNA and Cut&Tag sequencing performed in this study were deposited in the GEO database under accession code GSE224566 (https://www.ncbi.nlm.nih.gov/geo/query/acc.cgi?acc=GSE224566) and GSE210412 (https://www.ncbi.nlm.nih.gov/geo/query/acc.cgi?acc=GSE210412), respectively. The reference series is deposited under GSE224567 (https://www.ncbi.nlm.nih.gov/geo/query/acc.cgi?acc=GSE224567). The Clinical Proteomic Tumor Analysis Consortium (CPTAC) publicly available data used in this study are available in the LinkedOmics under pancreatic adenocarcinoma (http://www.linkedomics.org/data_download/CPTAC-PDAC/)37. The Cancer Genome Atlas (TCGA) publicly available data used in this study are available in the GDC Data Portal under TCGA-PAAD (https://portal.gdc.cancer.gov/projects/TCGA-PAAD)38. The remaining data are available within the Article, Supplementary Information or Source Data file. Source data are provided as a Source Data file.

Human research participants

Policy information about studies involving human research participants and Sex and Gender in Research.

Reporting on sex and gender	NA
Population characteristics	NA
Recruitment	NA
Ethics oversight	NA

Note that full information on the approval of the study protocol must also be provided in the manuscript.

Field-specific reporting

Please select the one below that is the best fit for your research. If you are not sure, read the appropriate sections before making your selection.

☒ Life sciences ☐ Behavioural & social sciences ☐ Ecological, evolutionary & environmental sciences

For a reference copy of the document with all sections, see nature.com/documents/nr-reporting-summary-flat.pdf

Life sciences study design

All studies must disclose on these points even when the disclosure is negative.

Sample size	No statical methods were used to calculate sample size. The sample size were determined based on the variation in pilot studies. The number of samples are described in each figure legends.
Data exclusions	No data were excluded from the analyses
Replication	The number of replication in each experiments are described in Figure legends and Method section. All attempts at replication were successful.
Randomization	Animals were randomly assigned to each arm of treatment and in vitro studies were randomly assigned to each group.
Blinding	For drug study in mice, we performed blinding test to evaluate the effect of the drugs. Data analysis and quantification were assigned to a group were not involved in the sample collection.

Reporting for specific materials, systems and methods

We require information from authors about some types of materials, experimental systems and methods used in many studies. Here, indicate whether each material, system or method listed is relevant to your study. If you are not sure if a list item applies to your research, read the appropriate section before selecting a response.

Materials & experimental systems

n/a	Involved in the study
☐	☒ Antibodies
☐	☒ Eukaryotic cell lines
☒	☐ Palaeontology and archaeology
☐	☒ Animals and other organisms
☒	☐ Clinical data
☒	☐ Dual use research of concern

Methods

n/a	Involved in the study
☐	☒ ChIP-seq
☐	☒ Flow cytometry
☒	☐ MRI-based neuroimaging

Antibodies

Antibodies used

Chicken polyclonal anti-GFP,Abcam Cat#ab13970,IHC 1:2000; Goat polyclonal anti-tdTomato,LifeSpan Cat#LS-C348313,IHC 1:2000; Ki67 Rabbit polyclonal antibody,MilliporeSigma Cat#ab9260,IHC 1:500; HNF-4 alpha/NR2A1 Rabbit polyclonal antibody,NOVUS BIOLOGICALS Cat#NBP1-89679,IHC 1:1000; CDH2 rat mAb,DSHB Cat#MNCD2,IHC 1:200; Mouse monoclonal Anti-E-Cadherin (clone36),BD Biosciences Cat#610182,IHC 1:2000; IF 1:500; WB 1:2000 ; Mouse monoclonal anti-Vinculin (Clone 7F9),Santa Cruz Biotechnology Cat#sc-73614,WB 1:2000; Mouse monoclonal anti-β-Actin (Clone C4),Santa Cruz Biotechnology Cat#sc-47778,WB 1:5000; ZEB1 (E2G6Y) XP Rabbit mAb,Cell Signaling Technology Cat#70512,IHC 1:500; JunB (C37F9) Rabbit mAb,Cell Signaling Technology Cat#3753,IHC 1:500; WB 1:1000; Rabbit monoclonal anti-c-Myc (D3N8F) ,Cell Signaling Technology Cat#13987,WB 1:1000; Rabbit polyclonal anti-YAP,Cell Signaling Technology Cat#4912,IHC 1:500; WB 1:1000; Cut&Tag 1:50; Rabbit monoclonal anti-SOX2 (Clone D6D9),Cell Signaling Technology Cat#5024,IHC 1:500; IF 1:200; WB 1:1000; Cut&Tag 1:50; SOX5 Polyclonal antibody,Proteintech Cat#13216-1-AP,IHC 1:500; IF 1:200; WB 1:1000; Cut&Tag 1:50; TWIST2 Polyclonal antibody,Proteintech Cat#11752-1-AP,IHC 1:500; IF 1:200; WB 1:1000; Cut&Tag 1:100; COUP-TFI (D4H2) Rabbit mAb antibody (NR2F1),Cell Signaling Technology Cat#6364,IHC 1:500; WB 1:1000; COUP-TFII (D16C4) Rabbit mAb (NR2F2),Cell Signaling Technology Cat#6434,IHC 1:500; WB 1:1000; ETS-1 (D8O8A) Rabbit mAb,Cell Signaling Technology Cat#14069,WB 1:1000 ; MAF polyclonal antibody,Proteintech Cat#55013-1-AP,WB 1:1000 ; SIX4 polyclonal antibody,Proteintech Cat#21305-1-AP,WB 1:1000 ; PRX (Prrx1; C-6) antibody,Santa Cruz Biotechnology Cat#sc-271047,WB 1:1000 ; p40 Mouse monoclonal antibody,BIOCARE MEDICAL Cat#ACI 3066 A,IHC 1:500; Rat monoclonal anti-Keratin, type I; cytokeratin 19,DSHB Cat#TROMA-III,IHC 1:500; WB 1:2000; Rabbit Anti-BRD4 antibody,Sigma-Aldrich Cat#HPA015055,Cut&Tag 1:50; Anti-acetyl-Histone H3 (Lys27) Antibody,MilliporeSigma Cat#07-360,Cut&Tag 1:50; Anti-trimethyl-Histone H3 (Lys4) Antibody,MilliporeSigma Cat#07-473,Cut&Tag 1:100; c-Jun (60A8) Rabbit mAb antibody,Cell Signaling Technology Cat#9165,WB 1:1000; Cut&Tag 1:100; Anti-rabbit IgG, HRP-linked Antibody ,Cell Signaling Technology Cat#7074,WB 1:5000; Anti-mouse IgG, HRP-linked Antibody,Cell Signaling Technology Cat#7076,WB 1:5000.

Validation

All information of each antibodies were available at each company website by cat# or RRID.
Chicken polyclonal anti-GFP, Abcam Cat#ab13970; RRID:AB_300798, Goat polyclonal anti-tdTomato, LifeSpan Cat#LS-C348313 (In 293HEK cells transfected with cds plasmid detects a band of 55 kDa by Western blot. It also detects tdTomato in brain sections by IHC.), Ki67 Rabbit polyclonal antibody, MilliporeSigma Cat#ab9260; AB_RRID:2142366, HNF-4 alpha/NR2A1 Rabbit polyclonal antibody, NOVUS BIOLOGICALS Cat#NBP1-89679 (Orthogonal Strategies Validation. Immunohistochemistry-Paraffin: HNF-4 alpha/ NR2A1 Antibody [NBP1-89679] - Staining in human duodenum and tonsil tissues using anti-HNF4A antibody. Corresponding HNF4A RNA-seq data are presented for the same tissues.), CDH2 rat mAb, DSHB Cat#MNCD2; RRID:AB_528119, Mouse monoclonal Anti-E-Cadherin (clone36), BD Biosciences Cat#610182; RRID:AB_397581, Mouse monoclonal anti-Vinculin (Clone 7F9), Santa Cruz Biotechnology Cat#sc-73614; RRID:AB_1131294, Mouse monoclonal anti-β-Actin (Clone C4), Santa Cruz Biotechnology Cat#sc-47778; RRID:AB_2714189, ZEB1 (E2G6Y) XP Rabbit mAb, Cell Signaling Technology Cat#70512 (Western blot analysis of extracts from various cell lines show 200kDa.), JunB (C37F9) Rabbit mAb, Cell Signaling Technology Cat#3753; RRID:AB_2130002, Rabbit monoclonal anti-c-Myc (D3N8F) , Cell Signaling Technology Cat#13987; RRID:AB_2631168, Rabbit polyclonal anti-YAP, Cell Signaling Technology Cat#4912; RRID:AB_2218911, Rabbit monoclonal anti-SOX2 (Clone D6D9), Cell Signaling Technology Cat#5024; RRID:AB_1904142, SOX5 Polyclonal antibody, Proteintech Cat#13216-1-AP; RRID:AB=2196089, TWIST2 Polyclonal antibody, Proteintech Cat#11752-1-AP; RRID:AB_2877791, COUP-TFI (D4H2) Rabbit mAb antibody (NR2F1), Cell Signaling Technology Cat#6364; RRID:AB_11220432, COUP-TFII (D16C4) Rabbit mAb (NR2F2), Cell Signaling Technology Cat#6434; RRID:AB_11220428, ETS-1 (D8O8A) Rabbit mAb, Cell Signaling Technology Cat#14069 (Western blot analysis of extracts from various cell lines using ETS-1 (D8O8A) Rabbit mAb show bands at 52kDa), MAF polyclonal antibody, Proteintech Cat#55013-1-AP; RRID:AB_10863127, SIX4 polyclonal antibody, Proteintech Cat#21305-1-AP; RRID:AB_10860258, PRX (Prrx1; C-6) antibody, Santa Cruz Biotechnology Cat#sc-271047; RRID:AB_10611937, p40 Mouse monoclonal antibody, BIOCARE MEDICAL Cat#ACI 3066 A; RRID:AB_2858274, Rat monoclonal anti-Keratin, type I; cytokeratin 19, DSHB Cat#TROMA-III; RRID:AB_2133570, Rabbit Anti-BRD4 antibody, Sigma-Aldrich Cat#HPA015055; RRID:AB_1845435", Anti-acetyl-Histone H3 (Lys27) Antibody, MilliporeSigma Cat#07-360; RRID:AB_310550, Anti-trimethyl-Histone H3 (Lys4) Antibody, MilliporeSigma Cat#07-473; RRID:AB_1977252, c-Jun (60A8) Rabbit mAb antibody, Cell Signaling Technology Cat#9165; RRID:AB_2130165, Anti-rabbit IgG, HRP-linked Antibody , Cell Signaling Technology Cat#7074, Anti-mouse IgG, HRP-linked Antibody, Cell Signaling Technology Cat#7076.

Eukaryotic cell lines

Policy information about cell lines and Sex and Gender in Research

Cell line source(s)

Panc1 (CRL-1469), 293T (CRL-3216) cells were purchased from ATCC. The other mice PDAC cell lines were established from GEMM. mT3 and mT4 PDAC cells lines were provided by Dr. David A. Tuveson.

Authentication

Authenticated cell lines were bought from ATCC. Primary PDAC cell lines were authenticated by genotyping PCR to compare with the corresponding mice.mT3 and mT4 mouse PDAC cell lines were authenticated by genotyping PCR to compare with the mice of origin, Kras+/LSL-G12D; Trp53+/LSL-R172H; Pdx1-Cre (KPC) model.

Mycoplasma contamination	Cells were mycoplasma negative by PlasmoTest Mycoplasma Detection Kit.
Commonly misidentified lines (See ICLAC register)	NA

Animals and other research organisms

Policy information about studies involving animals; ARRIVE guidelines recommended for reporting animal research, and Sex and Gender in Research

Laboratory animals	Genetically engineered mouse strains Yapflox/flox, TP53FRT/+, FSF-KrasG12D, R26CreER, R26Dual, and Pdx1-Flp were interbred to generate the experimental cohorts. NCr-nu/nu mice (strain; 553) or C57BL/6J were bought from NCI or Charles River Laboratories.
Wild animals	No wild animals were used in the study.
Reporting on sex	In the GEMM, gender of animals was not considered in the studies. The gender of GEMM used in the studies are following: #4449 (Female), #4455 (Female), #4516 (Male), #4610 (Female), #4641 (Female), #4697 (Female), #4785 (Male), #4808 (Female), #4852 (Male), #4891 (Female), #4892 (Female), #4921 (Female), #4938 (Female), #4943 (Male), #5024 (Female), #5136 (Male), #5346 (Male), #5376 (Male), #5379 (Male), #5462 (Female), #5520 (Male), #5522 (Male), #5596 (Female), #5601 (Male), #5720 (Female), #5897 (Male), #5996 (Male), #5997 (Female), #6028 (Female), #6032 (Male), #6055 (Female), #6114 (Female), #6134 (Male), #6139 (Female), #6149 (Female), #6365 (Male), #6385 (Male), #6387 (Female), #6389 (Female), #6394 (Male), #6484 (Female), #6516 (Male), #6547 (Female), #6597 (Male), #6749 (Female), #6821 (Male), #6928 (Female), #7369 (Male), #7641 (Female), #7783 (Female), #8224 (Male). There no difference in tumor progression by gender. For xenograft studies, we therefor used male NCr-nu/nu mice and C57BL/6J mice.
Field-collected samples	No field collected samples were used in the study.
Ethics oversight	All animal studies were conducted in compliance with ethical regulations according to protocol #2016-1192 approved by the Institutional Animal Care and Use Committee (IACUC) at Georgetown University.

Note that full information on the approval of the study protocol must also be provided in the manuscript.

ChIP-seq

Data deposition

☒ Confirm that both raw and final processed data have been deposited in a public database such as GEO.

☒ Confirm that you have deposited or provided access to graph files (e.g. BED files) for the called peaks.

Data access links<br>*May remain private before publication.*	The reference series is deposited under GSE224567<br>https://www.ncbi.nlm.nih.gov/geo/query/acc.cgi?acc=GSE224567<br><br>The subseries linked to GSE224567 are following;<br>https://www.ncbi.nlm.nih.gov/geo/query/acc.cgi?acc=GSE210412<br>To review GEO accession GSE210412<br>https://www.ncbi.nlm.nih.gov/geo/query/acc.cgi?acc=GSE224566<br>To review GEO accession GSE224566<br>Secure token: gvavuwuuvjozhsl
Files in database submission	GSE210412:<br>sm11.mm10.csaw.bw, sm133.mm10.csaw.bw, sm41.mm10.csaw.bw, sm67.mm10.csaw.bw, sm1.mm10.csaw.bw, sm136.mm10.csaw.bw, sm291.mm10.csaw.bw, sm3.mm10.csaw.bw, sm4.mm10.csaw.bw, sm5.mm10.csaw.bw, sm62.mm10.csaw.bw, sm87.mm10.csaw.bw, sm173.mm10.csaw.bw, sm182.mm10.csaw.bw, sm200.mm10.csaw.bw, sm210.mm10.csaw.bw, sm134.mm10.csaw.bw, sm304.mm10.csaw.bw, sm44.mm10.csaw.bw, sm70.mm10.csaw.bw, sm13.mm10.csaw.bw, sm26.mm10.csaw.bw, sm45.mm10.csaw.bw, sm71.mm10.csaw.bw, sm101.mm10.csaw.bw, sm22.mm10.csaw.bw, sm58.mm10.csaw.bw, sm84.mm10.csaw.bw, sm102.mm10.csaw.bw, sm51.mm10.csaw.bw, sm77.mm10.csaw.bw, sm94.mm10.csaw.bw, sm103.mm10.csaw.bw, sm19.mm10.csaw.bw, sm52.mm10.csaw.bw, sm78.mm10.csaw.bw, sm292.mm10.csaw.bw, sm294.mm10.csaw.bw, sm299.mm10.csaw.bw, sm36.mm10.csaw.bw, sm104.mm10.csaw.bw, sm23.mm10.csaw.bw, sm59.mm10.csaw.bw, sm85.mm10.csaw.bw, sm105.mm10.csaw.bw, sm37.mm10.csaw.bw, sm63.mm10.csaw.bw, sm7.mm10.csaw.bw, sm109.mm10.csaw.bw, sm39.mm10.csaw.bw, sm65.mm10.csaw.bw, sm9.mm10.csaw.bw, sm146.mm10.csaw.bw, sm152.mm10.csaw.bw, sm164.mm10.csaw.bw, sm169.mm10.csaw.bw, sm10.mm10.csaw.bw, sm40.mm10.csaw.bw, sm66.mm10.csaw.bw, sm88.mm10.csaw.bw, sm110.mm10.csaw.bw, sm16.mm10.csaw.bw, sm48.mm10.csaw.bw, sm74.mm10.csaw.bw, sm111.mm10.csaw.bw, sm24.mm10.csaw.bw, sm60.mm10.csaw.bw, sm86.mm10.csaw.bw, sm112.mm10.csaw.bw, sm25.mm10.csaw.bw, sm306.mm10.csaw.bw, sm61.mm10.csaw.bw, sm149.mm10.csaw.bw, sm155.mm10.csaw.bw, sm167.mm10.csaw.bw, sm172.mm10.csaw.bw, sm113.mm10.csaw.bw, sm20.mm10.csaw.bw, sm53.mm10.csaw.bw, sm79.mm10.csaw.bw, sm11.mm10.filtered.bam, sm133.mm10.filtered.bam, sm41.mm10.filtered.bam, sm67.mm10.filtered.bam, sm1.mm10.filtered.bam, sm136.mm10.filtered.bam, sm291.mm10.filtered.bam, sm3.mm10.filtered.bam, sm4.mm10.filtered.bam, sm5.mm10.filtered.bam, sm62.mm10.filtered.bam, sm87.mm10.filtered.bam, sm173.mm10.filtered.bam, sm182.mm10.filtered.bam, sm200.mm10.filtered.bam, sm210.mm10.filtered.bam, sm134.mm10.filtered.bam, sm304.mm10.filtered.bam, sm44.mm10.filtered.bam, sm70.mm10.filtered.bam, sm13.mm10.filtered.bam, sm26.mm10.filtered.bam, sm45.mm10.filtered.bam, sm71.mm10.filtered.bam,

sm101.mm10.filtered.bam, sm22.mm10.filtered.bam, sm58.mm10.filtered.bam, sm84.mm10.filtered.bam, sm102.mm10.filtered.bam, sm51.mm10.filtered.bam, sm77.mm10.filtered.bam, sm94.mm10.filtered.bam, sm103.mm10.filtered.bam, sm19.mm10.filtered.bam, sm52.mm10.filtered.bam, sm78.mm10.filtered.bam, sm292.mm10.filtered.bam, sm294.mm10.filtered.bam, sm299.mm10.filtered.bam, sm36.mm10.filtered.bam, sm104.mm10.filtered.bam, sm23.mm10.filtered.bam, sm59.mm10.filtered.bam, sm85.mm10.filtered.bam, sm105.mm10.filtered.bam, sm37.mm10.filtered.bam, sm63.mm10.filtered.bam, sm7.mm10.filtered.bam, sm109.mm10.filtered.bam, sm39.mm10.filtered.bam, sm65.mm10.filtered.bam, sm9.mm10.filtered.bam, sm146.mm10.filtered.bam, sm152.mm10.filtered.bam, sm164.mm10.filtered.bam, sm169.mm10.filtered.bam, sm10.mm10.filtered.bam, sm40.mm10.filtered.bam, sm66.mm10.filtered.bam, sm88.mm10.filtered.bam, sm110.mm10.filtered.bam, sm16.mm10.filtered.bam, sm48.mm10.filtered.bam, sm74.mm10.filtered.bam, sm111.mm10.filtered.bam, sm24.mm10.filtered.bam, sm60.mm10.filtered.bam, sm86.mm10.filtered.bam, sm112.mm10.filtered.bam, sm25.mm10.filtered.bam, sm306.mm10.filtered.bam, sm61.mm10.filtered.bam, sm149.mm10.filtered.bam, sm155.mm10.filtered.bam, sm167.mm10.filtered.bam, sm172.mm10.filtered.bam, sm113.mm10.filtered.bam, sm20.mm10.filtered.bam, sm53.mm10.filtered.bam, sm79.mm10.filtered.bam
GEO224566;
1_4269_GFP-63651621.bam, 2_4435_GFP-63649629.bam, 3_4504h_GFP-63657631.bam, 4_4504s_GFP-63650613.bam, 5_4515_GFP-63662614.bam, 6_4601_GFP-63658623.bam, 7_4449_Tm-63653618.bam, 11_4891_Tm-63665603.bam, 13_4938_Tm-63665604.bam, 1-4269-GFP_S140.read_counts.txt, 2-4435-GFP_S141.read_counts.txt, 3-4504h-GFP_S142.read_counts.txt, 4-4504s-GFP_S143.read_counts.txt, 5-4515-GFP_S144.read_counts.txt, 6-4601-GFP_S145.read_counts.txt, 7-4449-Tm_S146.read_counts.txt, 11-4891-Tm_S150.read_counts.txt, 13-4938-Tm_S151.read_counts.txt

Genome browser session (e.g. UCSC): No longer applicable

Methodology

Replicates: Experiments were performed with more than five biological replicates

Sequencing depth: At least 2M unique reads per each experiment, pair-ended

Antibodies: Anti-acetyl-Histone H3 (Lys27) Antibody, Millipore, Cat#07-360, RRID:AB_310550, Anti-trimethyl-Histone H3 (Lys4) Antibody, Millipore, Cat#07-473, RRID:AB_1977252, SOX5 Polyclonal antibody, Proteintech, Cat#13216-1-AP, RRID:AB_2196089, TWIST2 Polyclonal antibody, Proteintech, Cat#11752-1-AP, RRID:AB_2877791,c-Jun (60A8) Rabbit mAb antibody, Cell Signaling Technology, Cat#9165, RRID:AB_2130165, Rabbit monoclonal anti-SOX2 (Clone D6D9), Cell Signaling Technology, Cat#5024; RRID:AB_1904142, Rabbit-anti-YAP1,NOVUS Biologicals, Cat#NB110-58358

Peak calling parameters: -very-sensitive-local -no-mixed -dovetail -phred33 -X 1000 -interleaved

Data quality: -style factor -L 15 -localSize 150000 -fdr 0.00001

Software: TrimGalore (v 0.6.5), csaw (v3.15), DESeq2 (v3.15), Bowtie2 (v2.4.5), Picard tools (2.0.1), HOMER (v4.11), deepTools (v2.0), BEDtools (2.28.0),

Flow Cytometry

Plots

Confirm that:

☒ The axis labels state the marker and fluorochrome used (e.g. CD4-FITC).

☒ The axis scales are clearly visible. Include numbers along axes only for bottom left plot of group (a 'group' is an analysis of identical markers).

☒ All plots are contour plots with outliers or pseudocolor plots.

☒ A numerical value for number of cells or percentage (with statistics) is provided.

Methodology

Sample preparation: PDAC cells were dissociated with 0.25% trypsin, resuspended with medium and wash with 1xPBS twice.

Instrument: FACS LSRFortessa Cell analyzer

Software: FSC express 7

Cell population abundance: The purity of cells were over 98%.

Gating strategy: FSC-A and SSC-A gate to select all cells. FSC-H, FSC-W gate to isolate single cells. SYTOX blue gate to distinguish Live or dead cells. GFP and tdTomato gate to analyze the percentage within the cells.

☒ Tick this box to confirm that a figure exemplifying the gating strategy is provided in the Supplementary Information.
